# Unexpected consequences of bombing. Community level response of epiphytic diatoms to environmental stress in a saline bomb crater pond area

**DOI:** 10.1371/journal.pone.0205343

**Published:** 2018-10-25

**Authors:** Angéla Földi, Éva Ács, István Grigorszky, Luc Ector, Carlos Eduardo Wetzel, Gábor Várbíró, Keve Tihamér Kiss, Péter Dobosy, Zsuzsa Trábert, Andrea K. Borsodi, Mónika Duleba

**Affiliations:** 1 MTA Centre for Ecological Research, Sustainable Ecosystems Group, Tihany, Hungary; 2 Doctoral School of Environmental Sciences, Eötvös Loránd University, Budapest, Hungary; 3 MTA Centre for Ecological Research, Danube Research Institute, Budapest, Hungary; 4 University of Debrecen, Department of Hydrobiology, Debrecen, Hungary; 5 Environmental Research and Innovation Department (ERIN), Luxembourg Institute of Science and Technology (LIST), Belvaux, Luxembourg; 6 MTA Centre for Ecological Research, Danube Research Institute, Department of Tisza River Research, Debrecen, Hungary; 7 Eötvös Loránd University, Department of Microbiology, Budapest, Hungary; Fred Hutchinson Cancer Research Center, UNITED STATES

## Abstract

The spatial response of epiphytic diatom communities to environmental stress was studied in a moderately saline wetland area located in the plain of Danube-Tisza Interfluve, Hungary. The area is characterised by World War II bomb crater ponds and can be regarded as an excellent ecological model system where the dispersion of species is slightly limited by distance. To study the effect of environmental variables on the communities, canonical correspondence analysis was applied. Salinity, pH, total suspended solids, total phosphorous and depth proved to be significant environmental drivers in this analysis. The ecological status of the ponds was assessed with Ziemann’s halobity index, as the trophity-depending metric cannot be applied to these habitats (due to the naturally high phosphorus content). Ponds in “good” ecological status significantly differed from those appertaining to water quality category of “not-good” ecological status considering characteristic of natural astatic soda pans (e.g. salinity, pH, ammonium, total phosphorous concentration, nitrogen:phosphorous ratio and turbidity). The differences between epiphytic diatom communities inhabiting the ponds were detected using non-parametric multidimensional scaling. The samples formed three groups according to the types of ponds (“transparent”, “transitional” and “turbid”) based on the width of the macrophyte belt around them. Indicator species related to the ecological status of the ponds and diatom communities contributing to the separation of groups of ponds were identified. One of the indicator species differed from species already described. Light and scanning electron microscopy features and phylogenetic analyses based on three genes (18S and 28S rRNA genes, *rbc*L) proved that it was a new species of *Nitzschia* genus, closely related to *Nitzschia frustulum* and *Nitzschia inconspicua*. Therefore, description of a new species, *Nitzschia reskoi* Ács, Duleba, C.E.Wetzel & Ector is proposed. We concluded that the increasing abundance of *Nitzschia reskoi* was a signal of the degradation of the intermittent saline wetlands.

## Introduction

Global warming affects the structure, function and stability of lake ecosystems throughout the world [1]. Small, shallow ponds are especially vulnerable, but the habitat loss due to the contraction is also detectable in large shallow lakes [1]. In limnological studies, the small, shallow lakes, the intermittent lakes and puddles have not been highlighted against large lakes for a long time. Investigations over the last decades have pointed out that there are significant differences between the ecology of small and large lakes [2,3]. The small lakes are labile because of the low water volume; consequently, they rapidly indicate the change of the environmental conditions (e.g. climate change). Huge numbers of astatic soda pans have decreased during the last decades all over the world, especially in the Carpathian Basin due to human activity and global warming [4].

Saline lakes occur in every continent [5] and the total volume of water in these environments on a global scale nearly equals to that in freshwater lakes [6].

According to the chemical composition, there are two main types of inland high salinity lakes, the salted (dominated by sodium [Na^+^] and chloride [Cl^-^] ions) and the soda lakes (dominated by sodium, hydrocarbonate [HCO_3_^-^] and carbonate [CO_3_^2-^] ions). They can be also determined based on their hydrological cycles: perennial (or semiastatic) and astatic. Soda lakes are the most stable alkaline environments on Earth [7]. They form in arid and semiarid areas of tropical and subtropical deserts of North America, continental Asia, East African Rift Valley and small areas in Europe such as Kiskunság, in Hungary.

The soda water bodies on the Hungarian Great Plain have alkaline pH and generally lower salt content than seawater and other continental salt waters. The most characteristic ions are the Na^+^ and HCO_3_^-^, the other six ions K^+^, Ca^2+^, Mg^2+^, CO_3_^-^, Cl^-^, SO_4_^2-^ and other hydrological features of the soda pans show great variability. The daily changes of water temperature are rather high [8]. Polymixis, hypersalinity and high turbidity are also key factors in these waters [9]. Due to their highly stochastic environmental dynamics, the astatic saline waters have special but only partially discovered composition of biota. For example, a new trebouxiophycean picoalga (*Chloroparva pannonica* Somogyi, Felföldi & Vörös) was described in 2011 from this special environment, which is not only a new species but belongs to a new genus as well [10]. The winter bloom of picoeukaryotes in Hungarian astatic soda pans also served several undescribed species [11,12].

Ecological indicator species are living organisms that are easily monitored and whose status reflects or predicts the condition(s) of the environment where they are found, they have a narrow tolerance, a well-defined optimum and a high abundance [13]. Currently, benthic diatoms are applied as early warning indicators and signals for ecological problems, and they can serve as barometers for trends in ecological changes. Their usefulness in lentic environments has been already been proven. (e.g. [14–16]).

With the implementation of the European Water Framework Directive and recognition of the conservation values of the intermittent soda pans located in Hungary, a number of studies dealing with their benthic diatoms were published [17–21]. The diatom-based indices for assessing ecological status of the Hungarian astatic soda pans and perennial sodic lakes have been established [22]. However, to define the reference conditions and the effects of natural disturbances originated from anthropogenic impacts pose great challenges of the ecological status assessment, especially in these multi-stressed habitats. Here, diatom communities are very special and characteristic [18], frequently dominated by taxa of *Nitzschia* Hassal genus [23]. Species of the genus *Nitzschia* can be found in wide range of environments and pollution levels [24] and many of them are important bioindicators (e.g. [25,26]).

The accurate identification of species is important for water quality assessment [27]. The traditional approach for identification of diatoms is morphology-based using the features of their silica frustule. However, it requires trained taxonomists and is often challenged by the influence of life cycle stage and environment on valve morphology [27]. DNA-based methods have become more and more commonly used for improving identification and in biodiversity studies. One of the most crucial elements of these investigations is selecting the appropriate marker [28], which can be used for the identification of species (barcoding markers, [29]). The 18S rRNA gene (e.g. [30]) and 28S rRNA gene (e.g. [28]) and *rbc*L gene (e.g. [28,31]) are among the most intensively studied and were proposed as barcoding markers.

As a result of mistargeted bombing during the World War II, on some area in Hungary ponds with different diameters and depths were formed in the same time, close to each other. These habitats are good ecological model systems because the natural environmental stress can be studied, especially since the dispersion of species is slightly limited by distance [32,33].

Our goals were 1) to study the community level response of epiphytic diatoms to natural environmental stress in hyposaline/saline astatic ponds; 2) to examine the applicability of epiphytic diatoms in assessment of the ecological status of these special temporary wetlands; 3) to define indicator species contributing to the separation of ponds and indicate the good ecological status of the saline ponds.

One of the indicator species differed from the diatom species already described, therefore, we aimed 4) to provide its exact identification and relationships based on a detailed morphological description using scanning electron microscopy (SEM) and sequence information of three genes (18S rRNA, 28S rRNA and *rbc*L).

## Material and methods

### Ethics statement

Middle Danube Valley Inspectorate for Environmental Protection, Nature Conservation and Water Management granted the permission (number: KTVF:45737-4/2013) to carry out sampling in the area of the Kiskunság National Park, near to the village of Apaj for research purpose.

### Study area and sampling

A dense cluster of saline water bomb crater ponds (created by mistargeted bombing of the nearby airport during World War II) is situated in the northernmost part of the Kiskunság National Park (47°7.403'N 19°8.187'E), near to the village of Apaj, in the plain of Danube-Tisza Interfluve, Hungary. In an area of approximately 25 hectares (0.25 km^2^), 112 ponds with various size can be found, from which 48 were studied.

This area has patchy surface salinization, because the flow pattern of groundwater results in extensive surface salinization in those discharge areas where the infiltrating freshwater does not superimpose the upwelling saline water. The salts originate from the overpressured NaCl-type water of the Pre-Neogene basement and the NaHCO_3_-type water of the Neogene sediments [34]. In these conditons the ponds studied hold sodic water and they can be considered as models of sodic aquatic ecosystems.

As the sampling, the water chemical analysis and the light microscopy (LM) invstigation methods are described in detail in Vad et al. [33] and Ács et al. [23], here a short description is provided.

Benthic diatom samples were taken between 7 and 9 May 2014 within the framework of a project that aimed simultaneous sampling and investigation of multiple organism groups (benthic diatoms, zooplankton, macroinvertebrates and vertebrates). During the sampling physico-chemical variables were also measured *in situ* (water depth, Secchi depth and diameter of each pond, percentages of open water surface and macrophyte coverage, conductivity, pH and water temperature) or water samples were taken for laboratory analysis (total suspended solids, chlorophyll *a*, total phosphorus, ammonium and nitrate concentrations).

Benthic diatom samples (five replicates per pond) were primarily taken from green common reed (*Phragmites australis*) stems, or, if it was absent, from alkali bulrush (*Bolboschoenus maritimus*) or narrowleaf cattail (*Typha angustifolia*). A 10-cm section of the stems starting at 10 cm below the water surface was cut and and carried to laboratory where epiphyton was washed from stems into tap water using tooth brushes. Diatom frustules were cleaned with hydrogen-peroxide and hydrochloric acid and mounted with Naphrax mountant. Diatoms were identified under Olympus IX70 inverted microscope equipped with differential interference contrast (DIC) optics at magnification of 1500×.

For description of the new *Nitzschia* species 50 valves were measured and morphologically analysed.

### SEM investigations

For SEM observations the cleaned samples were filtered with additional deionized water through a 3-μm Isopore polycarbonate membrane filter (Merck Millipore). Filter was mounted on aluminium stub and coated with platinum using a Modular High Vacuum Coating System BAL–TEC MED 020 (BAL–TEC AG, Balzers, Liechtenstein) or with gold using rotary-pumped spatter coater Quorum Q150R S. Platinum-coated samples were investigated with an ultrahigh-resolution analytical field emission (FE) scanning electron microscope Hitachi SU–70 (Hitachi High-Technologies Corporation, Japan) operated at 5 kV and SEM images were taken using the lower (SE-L) and upper (SE-U) detector signal. Gold-coated samples were studied with Zeiss EVO MA10 SEM operated at 10 kV.

### Culturing and DNA analysis

For culturing, cells of the undescribed *Nitzschia* species were isolated using micromanipulator. The culture was established and maintained in WC medium [35] prepared from the water sample and incubated at 18°C with light:dark cycle of 12:12 hours.

The culture was filtered to planktonic mesh with 10 μm pore size and shaken into 10 mM Tris-HCl solution and then frozen until processing. To extract DNA, the sample was heated at 95°C for 15 min, then treated with glass beads using cell mill. After that proteins were digested using Proteinase K (recombinant, Fermentas) at 55°C for 3 h. From this crude lysate DNA was purified using the DNeasy Plant Mini Kit (Qiagen).

Polymerase chain reactions (PCR) were performed on the 18S rRNA, 28S rRNA and *rbc*L genes using the primers listed in Table 1. The PCR mixture contained the following components in the total volume of 25 μl: 1.25 U DreamTaq^TM^ DNA Polymerase (Thermo Scientific), 200 mM of each deoxinucleoside triphosphate (Fermentas), 1X DreamTaq Buffer (Thermo Scientific), 0.325 μM of each primer, and 1 μL of template. The PCR amplifications used the following heat protocol: initial denaturation at 98°C for 5 min, 32 cycles at 94°C for 1 min, 52–60°C (according to the applied primer pair) for 30 sec, 72°C for 1–1.5 min (according to the expected length of the product) and a final extension at 72°C for 10 min. The sequencing reactions and capillary electrophoreses were performed by Biomi Ltd.

**Table 1:** Primers used for amplification and sequencing of 18S rRNA and *rbc*L genes from the undescribed *Nitzschia* species.

Name	Marker	Sequence (5' to 3')	Reference
**1F**^**1**^^,^ ^**2**^^,^ ^**s**^	18S rRNA gene	AACCTGGTTGATCCTGCCAGT	[36]
**1528R**^**1**^	18S rRNA gene	TGATCCTTCTGCAGGTTCACCTAC	[36]
**Nit1113R**^**2**^^,^ ^**s**^	18S rRNA gene	GGAACCCAAAGACTTGTG	This study
**DPrbcL1**^**1**^	*rbc*L	AAGGAGAAATHAATGTCT	[37]
**DPrbcL7**^**1**^^,^ ^**2**^	*rbc*L	AARCAACCTTGTGTAAGTCTC	[37]
**Nit_rbcL583F**^**2**^^,^ ^**s**^	*rbc*L	GAAGGTTTAAAAGGTGG	This study
**Nit_lsu41F**^**1**^^,^ ^**2**^^,^ ^**s**^	28S rRNA gene	TAAGCATATAATTAAGCGG	This study
**Nit_lsu868R**^**2**^	28S rRNA gene	TGTACTCGCACATATG	This study
**Nit_lsu1068R**^**1**^^,^ ^**2**^^,^ ^**s**^	28S rRNA gene	ACGTCAGAATCGCTAC	This study

^1^ primers used in the first PCR

^2^ primers used in the second PCR

^s^ sequencing primers.

The final 18S rRNA gene (983 nt), 28S rRNA gene (708 nt) and *rbc*L gene (812 nt) sequences assembled from overlapping sequence fragments were searched in the NCBI GenBank using BLAST [38] to find sequences with the highest similarity, and then aligned to sequences downloaded from the database. The ribosomal gene sequences were aligned by secondary structure using the SILVA Incremental Aligner (SINA, [39] available from http://www.arb-silva.de/aligner/), the *rbc*L sequences were aligned by codon using the Clustal W implemented in MEGA 6 [40]. For phylogenetic analysis the substitution model was chosen from the models proposed by MEGA 6 software (‘Find best DNA models’ option) based on the Bayesian Information Criterion. The selected models were the Tamura–Nei model with gamma distribution and invariant sites [41] for 18S and 28S rRNA genes and generalised time-reversible (GTR) model with gamma distribution and invariant sites [42] for *rbc*L gene. Bayesian posterior probability of distribution was estimated using the Metropolis–coupled Markov Chain Monte Carlo (MCMC) as implemented in MrBayes 3.2 [43]. Uncorrected p-distance values were calculated with the MEGA 6.

### Ecological status assessment

The Hungarian ecological status assessment system uses Ziemann’s halobity index (H, [44]) for the astatic, small soda ponds (that belong to the biological type 3 in the Hungarian validated typology declared at 1155/2016 (III.31) Government decree). The value of the index is converted to a number between 1 and 20 using the following equation:

H=0.19x+1

(whereas 1 is the worst, 20 is the best value of the index). Detailed description of the method can be found in Ács et al. [22]. The boundary values of the index and Ecological Quality Ratio (EQR) are the followings: poor/bad: 2.7 (EQR: 0.2); moderate/poor: 5.3 (EQR: 0.4); good/moderate: 7.9 (EQR: 0.6); high/good: 14.3 (EQR: 0.8).

In the followings we use term “good” for ponds having high and good ecological status from sodic characteristics and “not-good” for ponds not reaching good status (moderate, poor or bad quality categories) that indicates the decreasing of sodic characteristics.

### Statistical analysis

The non-metric multidimensional scaling (NMDS) was applied to visualize differences in composition among sites with Bray-Curtis similarity index. The Indicator Species Analysis (IndVal; [45]) based on relative abundance values was applied to identify species that can be used to separate the groups and also to identify the indicator species of ponds having “good” ecological status, the calculation was made with ‘labdsv’ package in R environment. SIMPER (Similarity Percentage) method was used for visualising taxa being responsible for the observed difference between groups of samples [46]. The Bray-Curtis similarity measure was used in SIMPER calculation. As the data deviated significantly from normal distribution, Mann-Whitney test was carried out to compare the medians of the environmental and biological variables between the groups.

There was interest in whether the spatial variation or the environmental variables effects more of the diatoms communities, therefore we performed a variation partitioning amongst diatom communities using Moran’s eigenvector maps (MEM). For the MEM analysis 'varpart' and 'adespatial' [47] R packages according to suggestions were used [48,49].

The main environmental factors that drive community composition were identified by canonical correspondence analysis (CCA). To reduce the influence of rare taxa, only taxa occurring in at least 10% of studied ponds and having relative abundance minimum 1% in at least one pond (n = 24) were used.

Turbidity, Secchi depth and total suspended solids showed significant correlation (Pearson correlation coefficients were turbidity-total suspended solids: 0.92, Secchi depth-total suspended solids: -0.60, Secchi depth-turbidity: -0.64, p<0.05), therefore, only turbidity was used in the further analyses. Area and depth of ponds showed correlations with almost all of other environmental variables including emergent macrophyte coverage.

Relative abundance data were square-root transformed to normalize their distribution and reduce the influence of exceptionally dominant taxa. The environmental variables (except pH) were log transformed to normalize their distribution. The CCA, NMDS SIMPER and the Mann-Whitney test were calculated with software PAST version 3.0 [50].

The ponds were categorized according to the width of the surrounding macrophyte belt as follows:

1. wide (> about 30% of the diameter of pond)
2. narrow (< about 30% of the diameter of pond)
3. absent

Diatom communities were also characterized based on the following biological traits of the occurring species: cell size [51], oxygen requirement [52], nitrogen uptake (N-uptake) strategy [52] and a combined trait. Two traits are frequently used in trait-based studies of benthic diatoms [53]: i) combined traits and ii) cell size [51]. Furthermore, we considered two other traits, iii) oxygen requirement [52] and iv) N-uptake strategy [52] for the analysis. Values of these traits were obtained from the OMNIDIA 6.0.2 data base [54].

- Classification into combined trait categories is based on the ability of species to use nutrient resources and to resist to physical perturbation (S1 Table). Practically it is equivalent to adhering strategies of diatoms, and its categories were considered as guilds in the system proposed by Rimet & Bouchez [55] that is a modification of the system by Passy [56].
- According to the allometric theory [57], cell size—due to the surface to volume ratio—is the major determinant the specific physiological activities of algae [58] such as growth, nutrient uptake and light capture.
- Oxygen is produced by photosynthesis, the rate of which—and thus the oxygen production—is influenced by light, temperature and nutrient levels. In light-limited habitats diatoms may rely on heterotrophic metabolism [59] becoming oxygen consumer from producer. The used classification of oxygen requirements is based on the oxygen concentration of waters that species can tolerate [52].
- iv) Several studies have shown that diatoms are able to assimilate organic nitrogen compounds (e.g. [60]). The ability of heterotrophic nitrogen-uptake serves as an additional source of nitrogen for diatoms, particularly under nitrogen-poor conditions, which may occur in soda waters [61]. The sensitive nitrogen-autotrophic taxa can tolerate only very small concentrations of organically bound nitrogen, the tolerant nitrogen-autotrophic taxa can tolerate elevated concentrations of organic nitrogen. The facultative nitrogen-heterotrophic taxa need periodically elevated concentrations, while the obligate nitrogen-heterotrophic taxa need continuously elevated concentrations of organic nitrogen [52].

The species distribution is shown on a sketch map of Hungary prepared using the ESRI ArcView 10.2 GIS program.

### Nomenclature

The electronic version of this article in Portable Document Format (PDF) in a work with an ISSN or ISBN will represent a published work according to the International Code of Nomenclature for algae, fungi, and plants, and hence the new names contained in the electronic publication of a PLOS ONE article are effectively published under that Code from the electronic edition alone, so there is no longer any need to provide printed copies.

The online version of this work is archived and available from the following digital repositories: PubMed Central, LOCKSS.

## Results

### Physical-chemical and benthic diatom-based biological features of the ponds

The ponds showed considerable variation in their physical-chemical properties (Table 2). All of them were alkaline, their trophic conditions varied from oligotrophic to hypertrophic. The salinity of the ponds varied from 0.97 to 5.5 g L^-1^, the Secchi transparency and turbidity varied in large intervals, the electric conductivity was 3.4 mS cm^-1^ in average (Table 2).

**Table 2:** Mean and ranges values of the environmental variables recorded in three groups of the 48 investigated bomb crater ponds.

Environmental variables	Abbr. on figs and in text	Mean (min-max) in "transparent" group	Mean (min-max) in "transitional" group	Mean (min-max) in "turbid" group
**Area (m**^**2**^**)**		29.8 (7.1–50.2)	42.5 (12.6–70.8)	60.6 (28.3–86.5)
**Depth (cm)**	dep	33.9 (15–53)	41.2 (14–60)	24.6 (4–38)
**Salinity (g L**^**-1**^**)**	Sal	1.7 (1.0–2.5)	2.8 (1.4–4.0)	3.4 (2.9–3.8)
**Electric conductivity (mS cm**^**-1**^**)**		2.2 (1.2–3.3)	3.6 (1.8–5.1)	4.4 (3.8–4.9)
**pH**		8.1 (7.8–8.4)	8.5 (8.0–9.0)	8.7 (8.2–9.0)
**Secchi depth (cm)**		26.1 (15–53)	17.6 (4–50)	8.4 (4–15)
**Turbidity (NTU)**	Turb	28.9 (4.8–97.0)	124.8 (22–691)	245.7 (72–539)
**Total suspended solids (mg L**^**-1**^**)**	TSS	27.8 (6.8–91.2)	83.5 (14.2–372)	143.9 (64.3–388)
**Total phosphorus (μg L**^**-1**^**)**	TP	91.7 (32.7–221.2)	213.4 (45.2–690.3)	838.0 (255.0–1693.6)
**Chlorophyll *a* (μg L**^**-1**^**)**	Chl	10.2 (4.9–14.3)	32.7 (0–387.6)	34.5 (8.8–131.4)
**Nitrate-N (mg L**^**-1**^**)**	nit	0.35 (0.13–0.83)	0.36 (0.15–0.73)	0.21 (0.13–0.32)
**Ammonium-N (mg L**^**-1**^**)**	amm	0.11 (0.01–0.35)	0.37 (0.01–3.51)	0.37 (0.03–1.01)
**Open water surface (%)**		73.2 (40–96)	89.6 (50–99.5)	91.3 (80–99)
**Submerged macrophyte coverage (%)**		0.1 (0–1)	1.3 (0–20)	0.6 (0–3)
**Emergent macrophyte coverage (%)**		26.8 (3–60)	9.1 (0.5–50)	6.7 (1–10)

**Fig 1:**
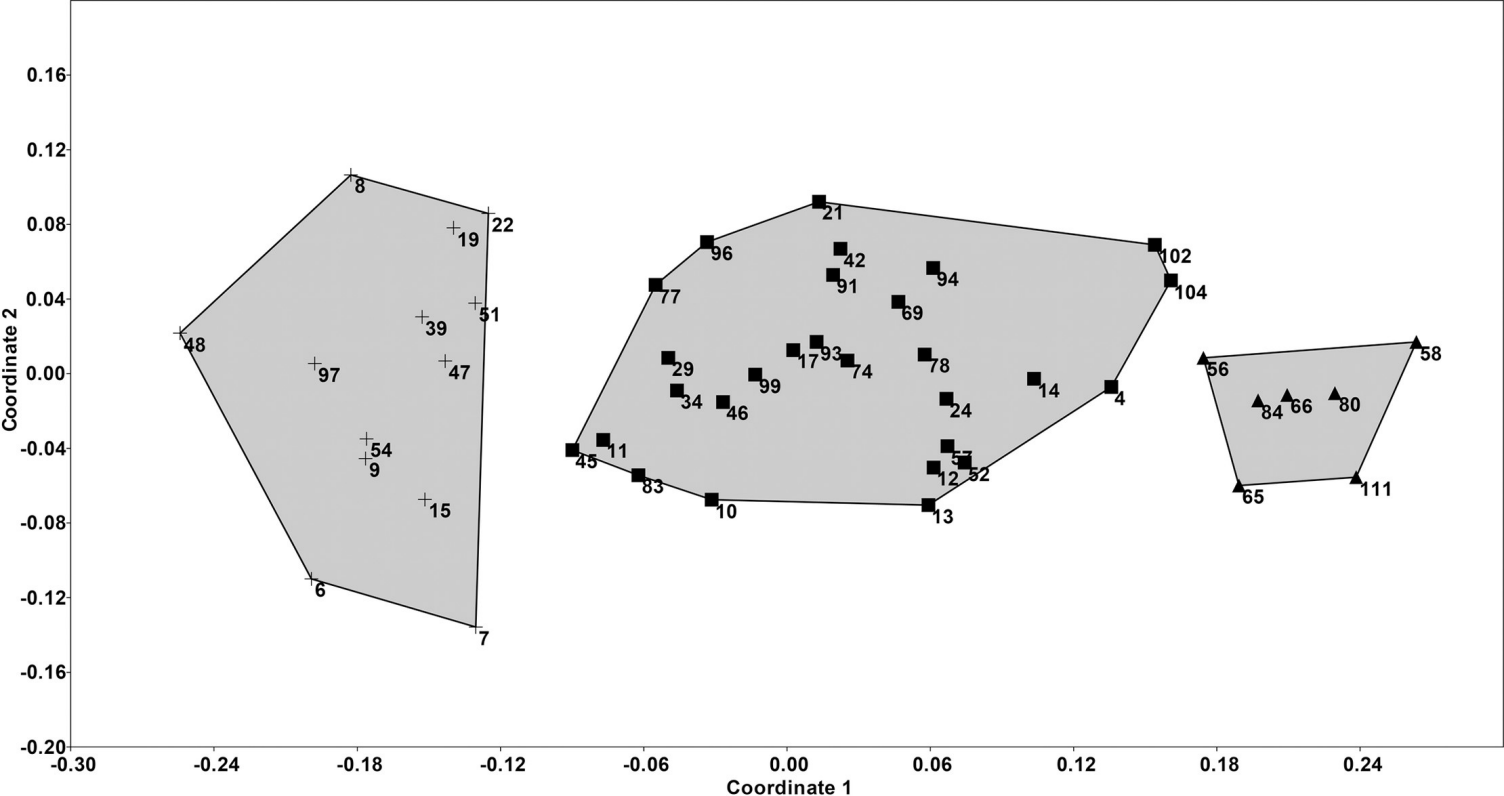
Non-metric multidimensional scaling (NMDS) ordinations of diatom communities. The marks indicate the macrophyte belt categories: cross = “transparent” group, filled square = “transitional” group, filled triangular = “turbid” group.

The differences in physical and water chemical variables enabled different benthic diatom communities to develop in the ponds. The NMDS showed that the ponds formed three distinct groups (Fig 1). The results of the NMDS met to the macrophyte belt categories for this study: 1: ponds with wide macrophyte belts, are refered to as “transparent” group (Part A in S1 Fig); 2: ponds with narrow macrophyte belts, are refered to as “transitional” group (Part B in S1 Fig); 3: ponds without macrophyte belts, are refered to as “turbid” group (Part C in S1 Fig).

**Fig 2:**
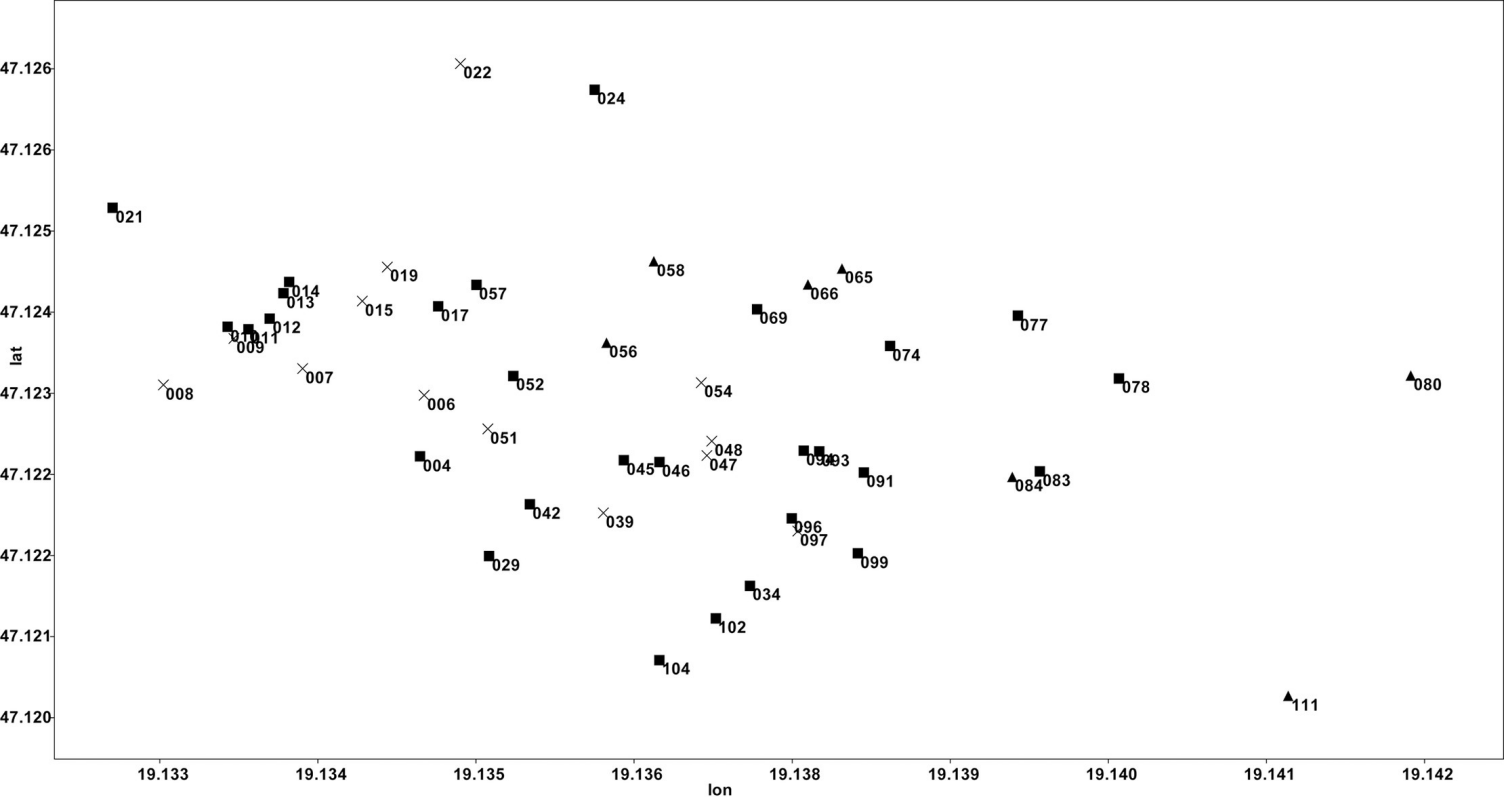
Location of the ponds in latitude-longitude coordinate system. The marks indicate the macrophyte belt categories: cross = “transparent” group, filled square = “transitional” group, filled triangular = “turbid” group.

There was no systematic spatial pattern in the physical-chemical variables of the ponds belonging to different groups, and the diatom composition was also quite different (Fig 2), as they exhibited mosaic-like arrangements.

The effect of spatiality was tested with Moran’s eigenvector maps (MEM) analysis, which shows that the spatial component compared to the environmental parameters has a negligible effect of the studied diatoms communities. (Env adj R2: 0.401. compared to Space adj. R2: 0.018).

The “transparent” group contained the ponds having low salinity, pH, TP and high transparency (Table 2). These ponds had the smallest area, and they were surrounded with wide macrophyte belt. The “transitional” group included the ponds having higher salinity, pH, TP, nitrate and ammonium content but moderate transparency (moderate suspended matter content). The “turbid” group involved ponds having the highest salinity, pH, TP, turbidity, elevated chlorophyll *a* content and they have the largest area.

### Ecological status of ponds based on benthic diatoms

Of the 48 studied ponds 18 were in good or high status based on benthic diatoms (S2 Table). The majority of the ponds having “good” status (11) belonged to the “transitional” group, the remaining belonged to the “turbid” group. Almost every pond belonging to the “turbid” group was in “good” status.

**Fig 3:**
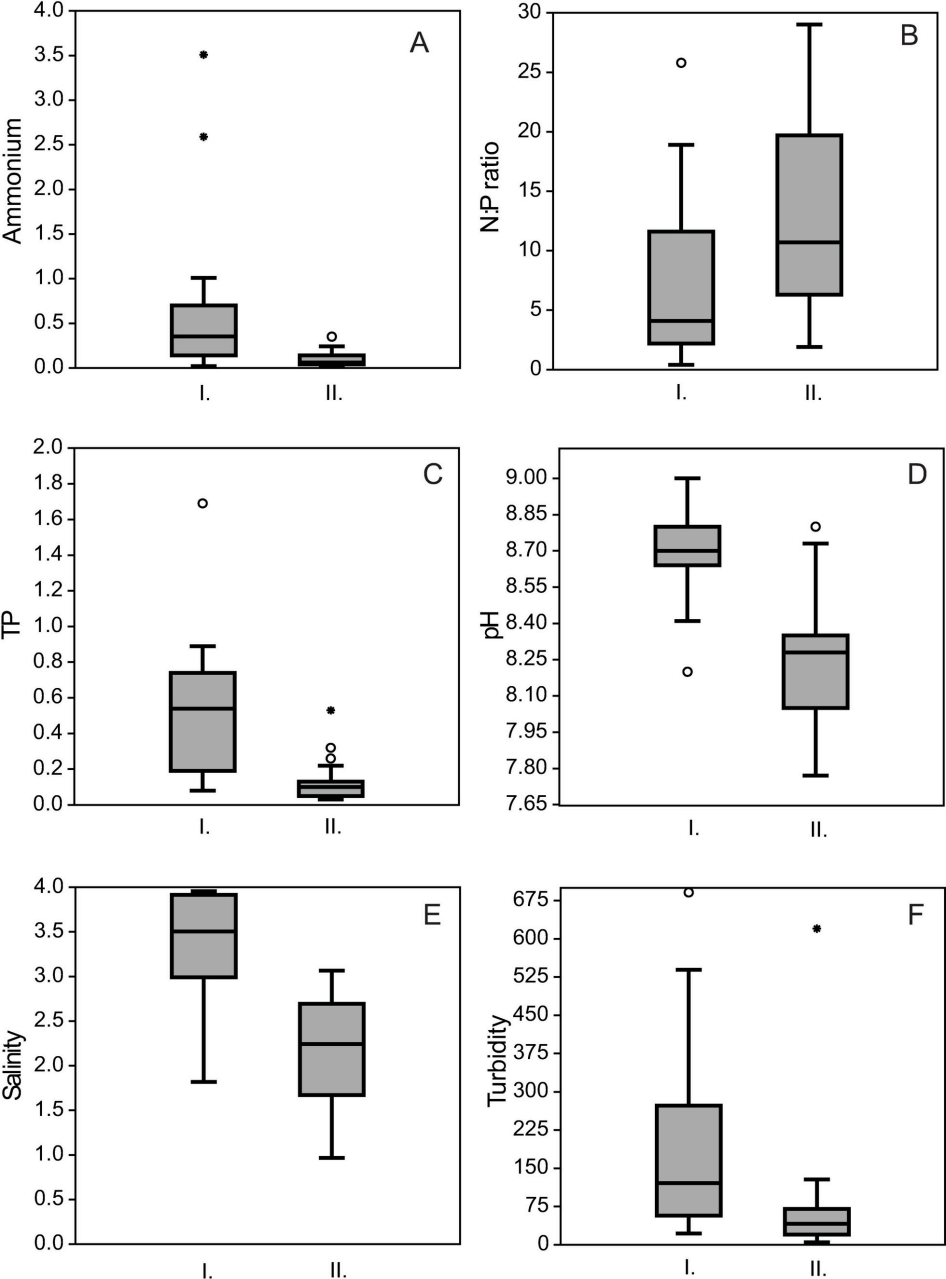
Box-plots showing the physical and chemical differences between the ponds in “good” and “not-good” ecological status. All differences were significant. Lines represent the medians, boxes represent the interquartile ranges (25–75%), whiskers represent 1.5 interquartile ranges, stars and circles represent outliers. A: ammonium ion concentration (mg L^-1^), B: nitrogen:phosphorous ratio, C: total phosphorous (mg L^-1^), D: pH, E: salinity (g L^-1^), F: turbidity (NTU). I = “good”, II = “not-good”.

**Fig 4:**
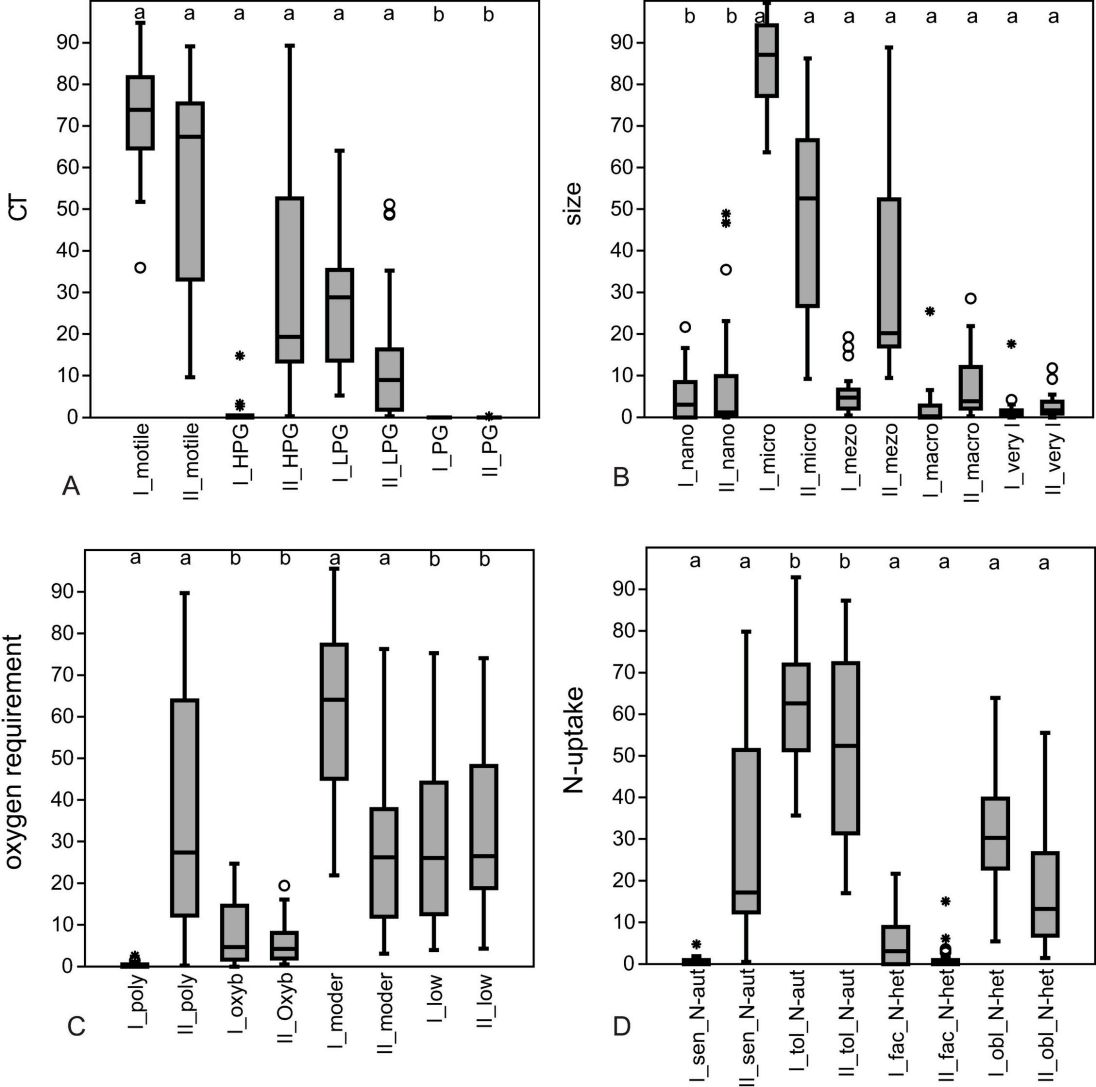
Box-plots showing the differences between relative abundances of categories of traits found in the ponds in “good” and “not-good” ecological status. A: combined trait, B: cell size, C: oxygen requirement, D: nitrogen uptake strategy. Within each traits a: significant, b: non-significant differences. Lines represent the medians, boxes represent the interquartile ranges (25–75%), whiskers represent 1.5 interquartile ranges, stars and circles represent outliers. I = “good”, II = “not-good”.

According to the Mann-Whitney test the salinity, pH, ammonium, TP, nitrogen:phosphorous (N:P) ratio and turbidity differed significantly between ponds in “good” and in “not-good” status (Fig 3), other measured chemical variables showed no significant differences. Turbidity, pH, salinity, total phosphorous and ammonium ion content were higher in ponds in “good” status, however, the N:P ratio was lower than in ponds in “not-good” status. The relative abundances of motile or adnate diatoms having micro (100 ≤ 300 μm^3^) cell size, moderate oxygen requirements and heterotrophic N-uptake strategy (facultative or obligate) were significantly higher in ponds in “good” status. The relative abundances of polyoxybiont, sensitive N-autotrophic, erected diatoms with meso (300 ≤ 600 μm^3^), macro (600 ≤ 1500 μm^3^) and large (>1500 μm^3^) cell size were significantly higher (Fig 4) in ponds in “not-good” status.

**Fig 5:**
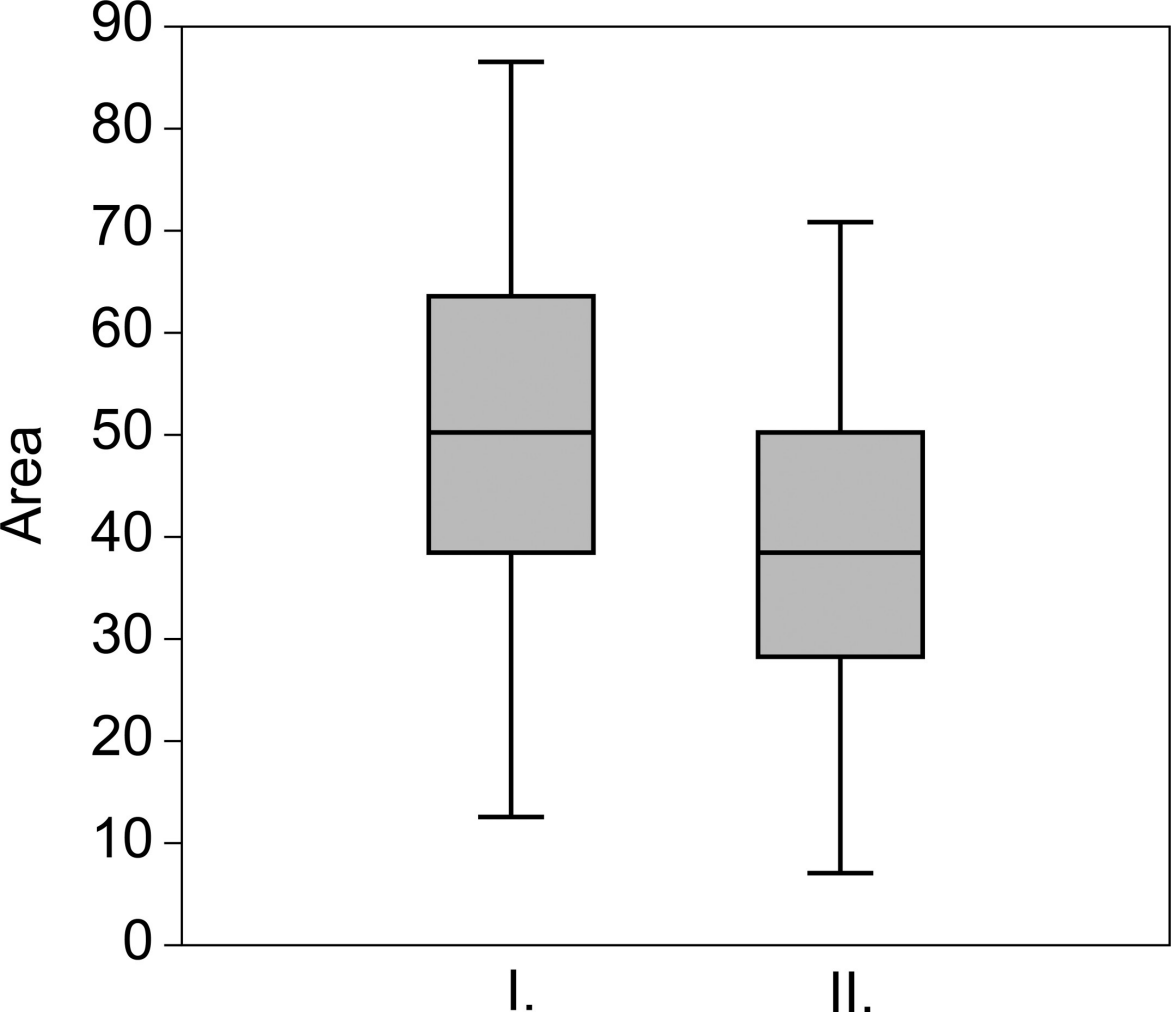
Box-plots showing the area differences of ponds in “good” and “not-good” ecological status. The difference was significant. Lines represent the medians, boxes represent the interquartile ranges (25–75%), whiskers represent 1.5 interquartile ranges. I = “good”, II = “not-good”.

Ponds in “good” status had significantly higher area (Fig 5), however, they were exposed strongly to the wind (they were surrounded by no or just narrow macrophyte belt).

### Main drivers of the diatom composition

**Fig 6:**
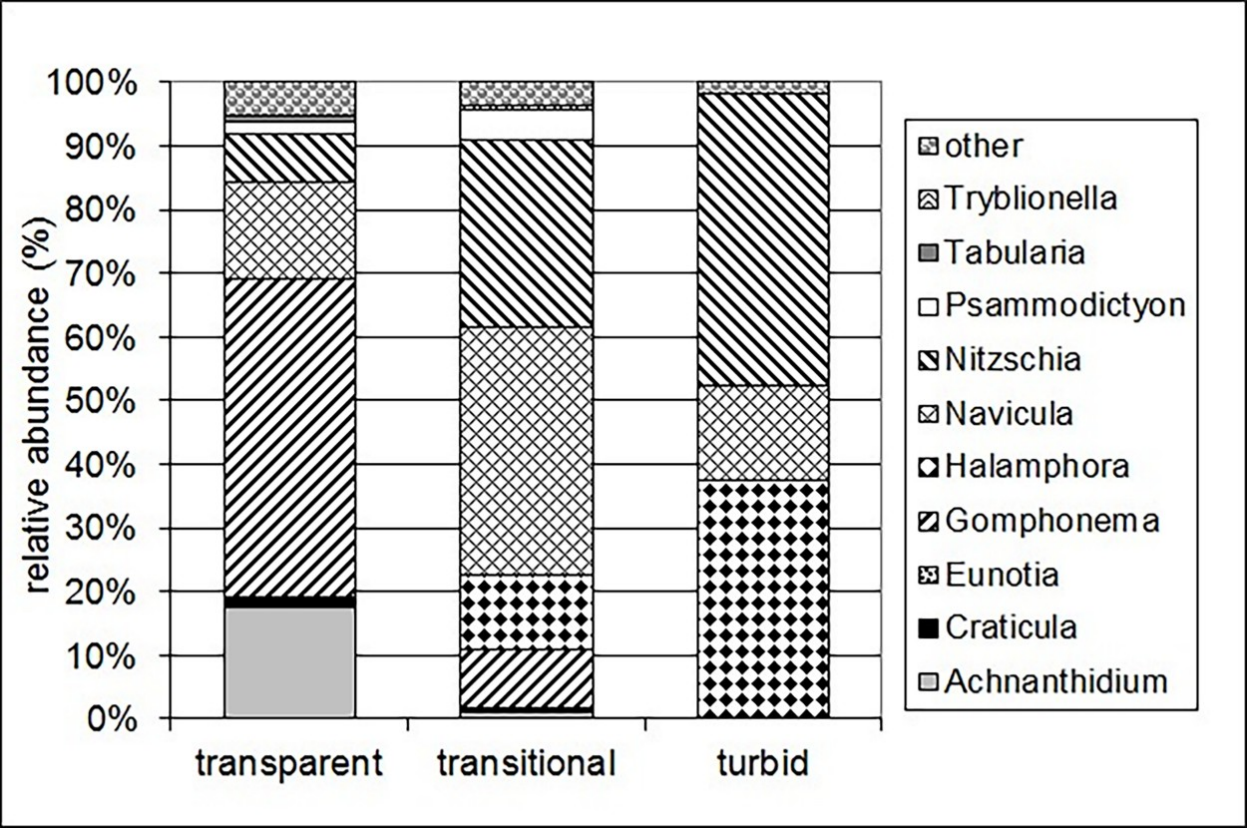
The proportion of the relative abundances of the dominant diatom genera in the three groups.

Altogether 80 diatom taxa, representing 33 genera, were found in this study. All taxa were identified to species level with the exception of four taxa. The three taxa were identified to genus level. The group of centrics were treated separately because it contained small, planktic organisms that were difficult to identify under light microscope. This group occurred only in one sample in a small amount. Twenty-four species were dominant (their relative abundance reached 5% in at least one sample) in the ponds (S3 Table). In the “transparent” group the genus *Gomphonema* had the highest proportion, it was followed by the genus *Achnanthidium*, while in the “transitional” and “turbid” groups the genera *Halamphora*, *Navicula* and *Nitzschia* dominated the epiphyton (Fig 6). The frequency of the most dominant species exceeded 20% (it means that they occurred in more than ten ponds). In addition, the following species had more than 20% frequency: *Anomoeoneis sphaerophora* (Ehrenberg) E.Pfitzer, *Craticula ambigua* (Ehrenberg) D.G.Mann, *Nitzschia commutata* Grunow in Cleve & Grunow, *Nitzschia vitrea* Norman, *Pseudofallacia monoculata* (Hustedt) Yan Liu, Kociolek & Quanxi Wang. The most frequent species were *Navicula veneta* Kützing and an unidentified *Nitzschia* species occurring in every studied pond. The mean of their relative abundances reached the highest values in the “transparent” group.

**Fig 7:**
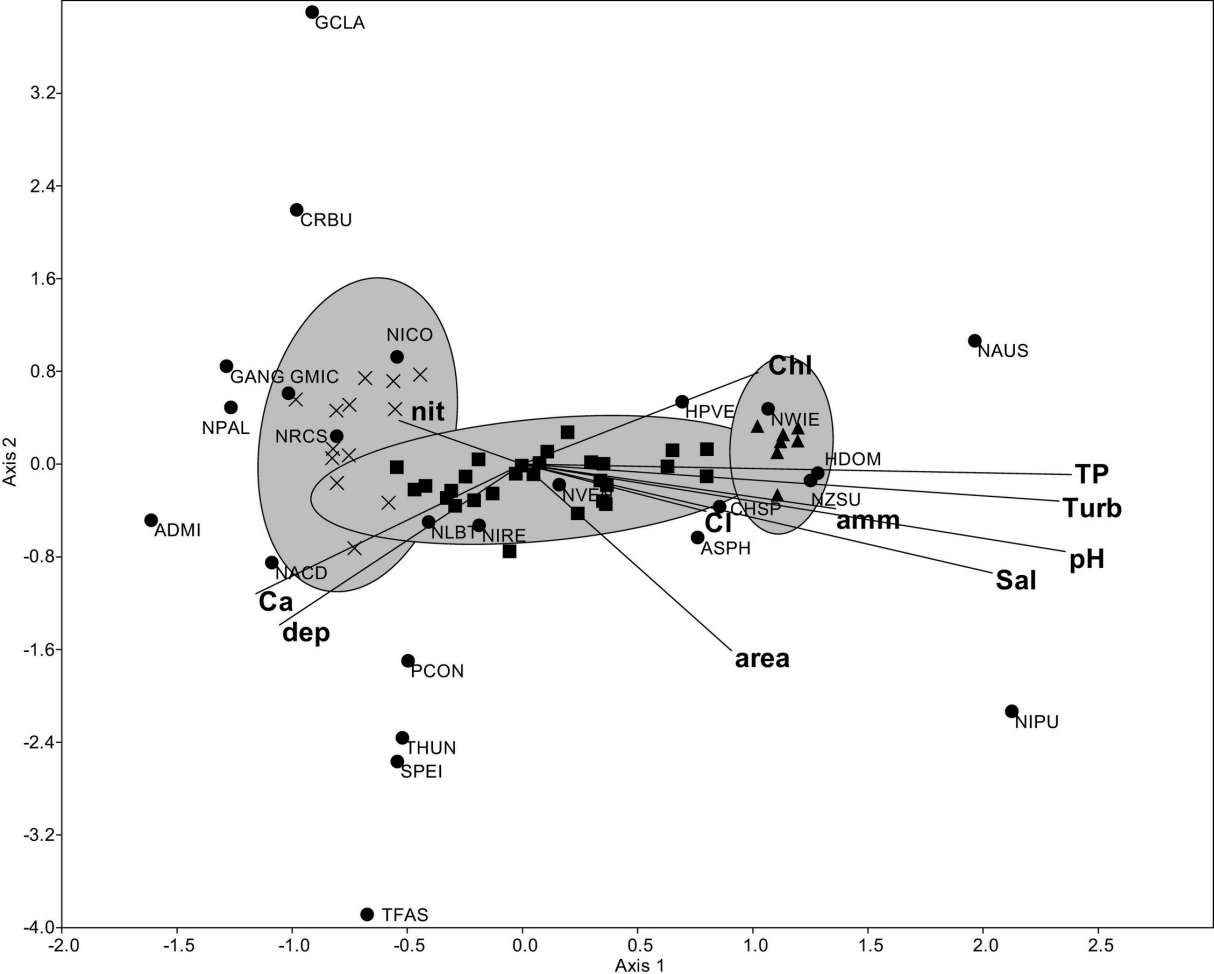
Canonical correspondence analysis showing the relationships between the relative abundances of the dominant diatom taxa and the environmental variables plotted with 95% ellipses. Environmental variables are indicated with lines, while species are indicated with points. Cross = samples from “transparent”, filled square = “transitional” and filled triangular = “turbid” ponds. The percentage of explained variance of axis 1 is 56.02 and that of axis 2 is 12.85. ASPH = *Anomoeoneis sphaerophora*, CHSP = *Chamaepinnularia* sp., NICO = *Nitzschia commutata*, NPAL = *Nitzschia palea* (Kützing) W.Smith var. *palea*, SPEI = *Surirella peisonis* Pantocsek. See other abbreviations in S2 and S3 Tables.

Salinity, pH, TSS, TP and depth all proved to be significant environmental drivers of the community composition based on the CCA analysis. *Halamphora dominici* Ács & Levkov, *Nitzschia pusilla* (Kützing) Grunow emend Lange-Bertalot, *Nitzschia austriaca* Hustedt and *Nitzschia supralitorea* Lange-Bertalot were positively correlated with salinity, pH, TSS and TP, while *Craticula accomoda* (Hustedt) Mann and *Psammodictyon constrictum* (Gregory) D.G. Mann in Round & al. with water depth. *Achnanthidium minutissimum* (Kützing) Czarnecki, *Gomphonema angustatum* (Kützing) Rabenhorst and *Nitzschia palea* (Kützing) W.Smith var. *palea* proved to be exceptionally dominant in waters with low salinity, pH, TP and TSS values (Fig 7).

### Indication

The IndVal analysis identified 15 indicator species with significant indicator values for the bomb crater ponds (Table 3); six of them belonged to the “transparent”, four to the “transitional” and five to the “turbid” groups.

**Table 3:** The most significant indicator species based on IndVal.

Taxon	Group	Indicator value	Probability
*Gomphonema angustatum* (Kützing) Rabenhorst	“transparent”	0.8682	0.001
*Achnanthidium minutissimum* (Kützing) Czarnecki	“transparent”	0.6551	0.003
*Gomphonema clavatum* Ehrenberg	“transparent”	0.6465	0.002
*Gomphonema* cf. *micropus* Kützing var. *micropus*	“transparent”	0.5136	0.03
*Gomphonema affine* Kützing	“transparent”	0.3077	0.009
*Gomphonema paludosum* Reichardt	“transparent”	0.3077	0.006
*Nitzschia* cf. *liebetruthii* Rabenhorst var. *liebetruthii*	“transitional”	0.7232	0.001
*Psammodictyon constrictum* (Gregory) D.G. Mann in Round & al.	“transitional”	0.6326	0.009
**the newly described *Nitzschia* species**	“transitional”	0.6253	0.002
*Navicula veneta* Kützing	“transitional”	0.5766	0.001
*Nitzschia austriaca* Hustedt	“turbid”	0.9013	0.001
*Halamphora dominici* Ács & Levkov	“turbid”	0.7404	0.001
*Nitzschia supralitorea* Lange-Bertalot	“turbid”	0.6775	0.002
*Navicula wiesneri* Lange-Bertalot	“turbid”	0.5984	0.006
*Navicula radiosa* Kützing	“turbid”	0.2857	0.014

The Indicator Species Analysis also showed that four species namely *Halamphora dominici*, *Navicula wiesneri* Lange-Bertalot, *Nitzschia austriaca*, *Nitzschia supralitorea* indicated “good” ecological status (in SIMPER analysis they showed 3.5–25.5% contribution), while nine species were significantly related to the “not-good” status. The latter ones were *Achnanthidium minutissimum*, *Craticula buderi* (Hustedt) Lange-Bertalot, *Gomphonema angustatum*, *Gomphonema* cf. *micropus* Kützing, *Gomphonema clavatum* Ehrenberg, *Nitzschia acidoclinata* Lange-Bertalot, *Nitzschia commutata*, *Nitzschia palea* (Kützing) W.Smith var. *palea* and *Psammodictyon constrictum* (they showed 0.117–23% contribution in SIMPER analysis). The newly described *Nitzschia* species was not an indicator species in ponds in “good” or ponds in “not-good” status.

### Identification of the new *Nitzschia* sp

Based on the IndVal Analysis, one of the indicator species of the “transitional” group was a *Nitzschia* species that we could not identify exactly with LM. Therefore, a detailed SEM study was performed on it. It differed from other known diatoms in micromorphological features. Therefore, a pure culture has been established, and sequence analysis of three different DNA regions was carried out.

#### Microscopical investigations

**Fig 8:**
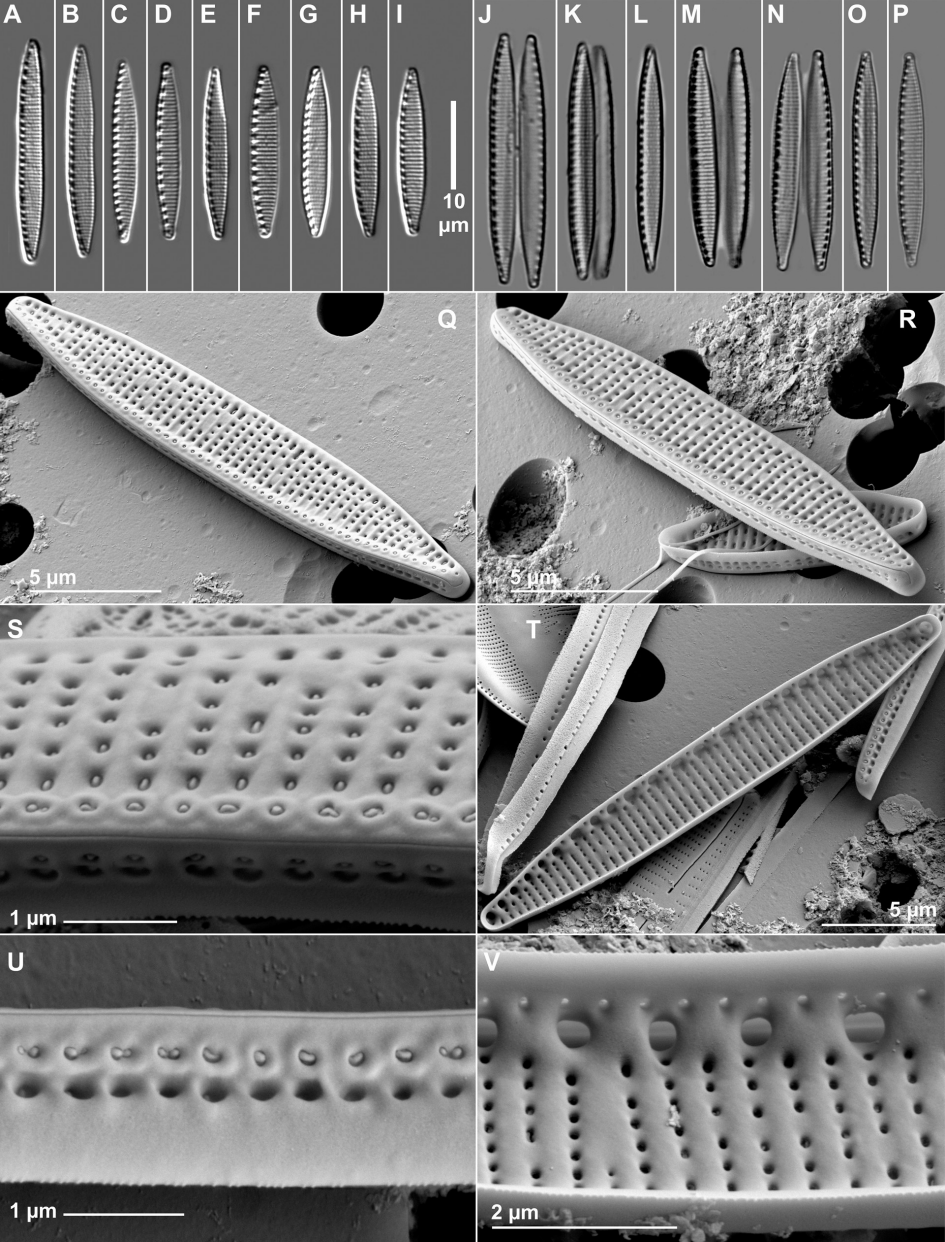
LM (A-P) and SEM (Q-V) micrographs of the new taxon isolated from Apaj.

The shape of valves is lanceolate with slightly protracted, narrow apices ends (Fig 8A–8P). Transapical striae are visible and the areolae can be resolved in LM. The fibulae are regularly distributed along the raphe canal. SEM features externally: the striae are uniseriate, both on the valve face and within the raphe canal, where each stria is represented by a single areola. The areolae are generally circular, sometimes irregular, some of them missing and the last areola is transapically elongated near the margin, opposite to raphe canal (Fig 8Q and 8R). Every first areolae near the raphe canal have a thickened ring around them. On the mantle, the striae are very short, comprising two areolae (Fig 8S and 8U), one of them cannot internally be detected. Raphe is continuous. Distal raphe fissures hooked (Fig 8Q and 8R). Internal distal raphe endings terminating in helictoglossae (Fig 8T). Internally, canal raphe subtended by fibulae widening to the valve face to form round portulae. Fibulae more or less evenly spaced throughout the valve, their shape is rectangular, one fibula is connected to two-three striae (Fig 8T). The raphe slit is uninterrupted, so it has no central nodule (Fig 8V).

#### DNA analysis

According to the BLAST search, sequences of our taxon showed the highest similarity with the species of *Nitzschia* section Lanceolatae: *Nitzschia inconspicua* Grunow, *N*. *amphibia* Grunow, *N*. *hantzschiana* Rabenhorst, *N*. *frustulum* (Kützing) Grunow in Cleve & Grunow (Table 4). These species are among the taxa to which the new species is morphologically the most similar.

**Table 4:** The results of the BLAST search.

Sequence of the new species	The most similar sequences	Identity
**18S rRNA gene** **(MG888433)**	*Nitzschia inconspicua* clone TCC487 (KC736636.1)	964/977(99%) with 4 gaps*
	*Nitzschia amphibia* isolate TCC574 (KT072977.1), *Nitzschia hantzschiana* isolate TCC510 (KT072967.1)	968/985(98%) with 4 gaps
**28S rRNA gene** **(MG888434)**	*Nitzschia inconspicua* strain G1_2 (HF679152.1)	662/713(93%) with 6 gaps
	*Nitzschia inconspicua* strain G3_4 (HF679162.1), *Nitzschia inconspicua* strain G3_2 (HF679160.1)	664/714(93%) with 6 gaps
***rbc*L** **(MG888435)**	*Nitzschia inconspicua* clone TCC481 (KC736606.1)	779/809(96%) with no gaps*
	*Nitzschia frustulum* strain Nit24 (HF675069.1)	778/809(96%) with no gaps*

The table shows the records that reached the two highest maximum scores. Identity is expressed in the rate of the identical sites to the compared sites.

*Comparisons in which our sequence could not be fully aligned to that in the database (coverage = 99%).

NCBI GenBank accession numbers are in brackets.

**Fig 9:**
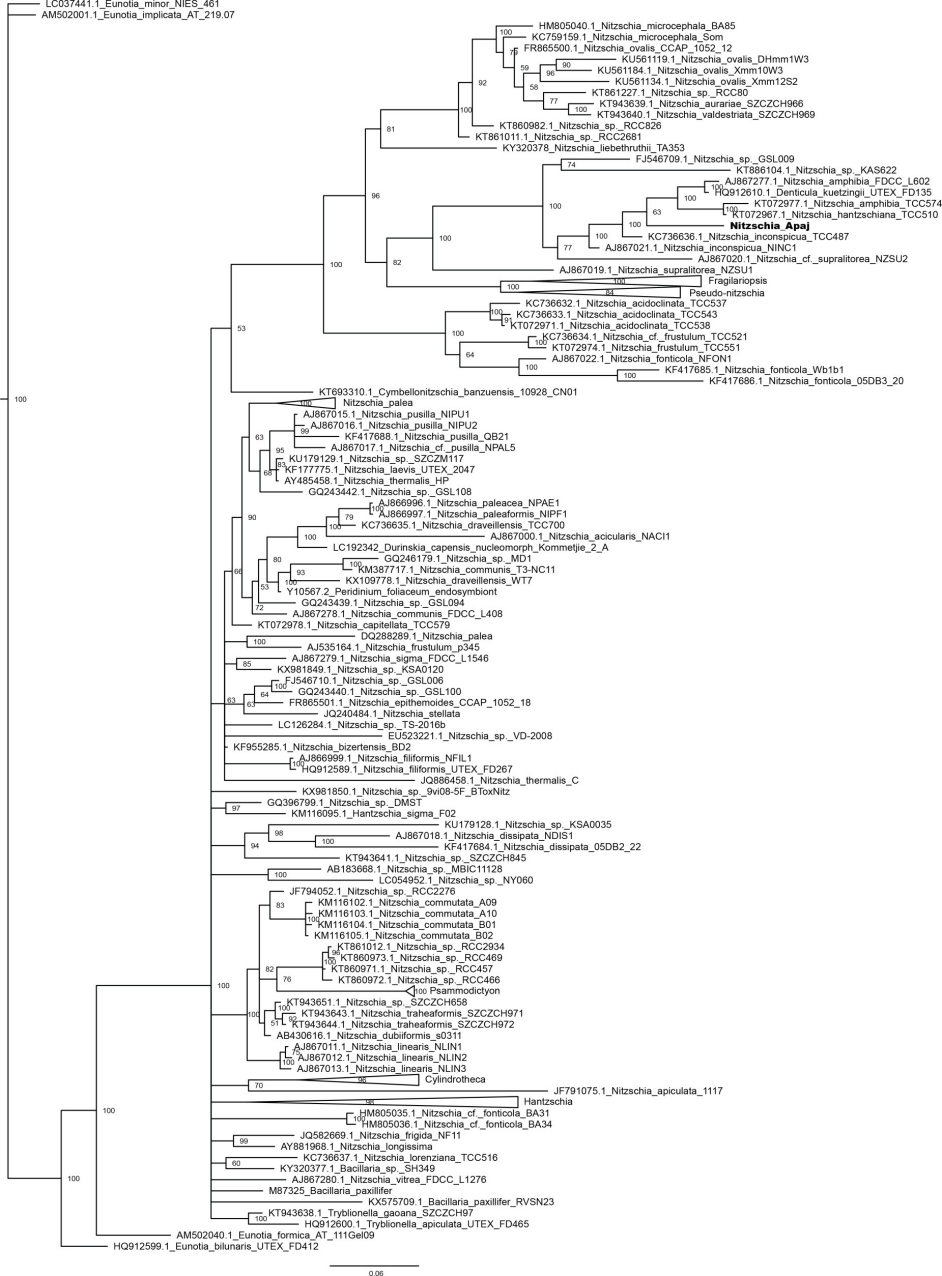
Bayesian inferred phylogenetic tree based on 18S rRNA gene sequences. For clarity certain clades were collapsed to triangles. Posterior probability values are indicated at the nodes. Sequence acquired in this study is indicated with bold letters. Scale bar: 0.06 substitutions/site.

On the 18S rRNA gene based phylogenetic tree (Fig 9) the new taxon formed a clade with *Nitzschia inconspicua*, *N*. *amphibia*, *N*. *hantzschiana*, and *Denticula kuetzingii* Grunow, however, the former two species did not seem to be monophyletic. The former species grouped together with a monophyletic clade of the isolates of *N*. *supralitorea* and formed the sister group of *Fragilariopsis* and *Pseudo*-*nitzschia*. *Nitzschia liebetruthii* Rabenhorst var. *liebetruthii* as well as *Nitzschia frustulum* and *N*. cf. *frustulum* were located in the next two lineages.

**Fig 10:**
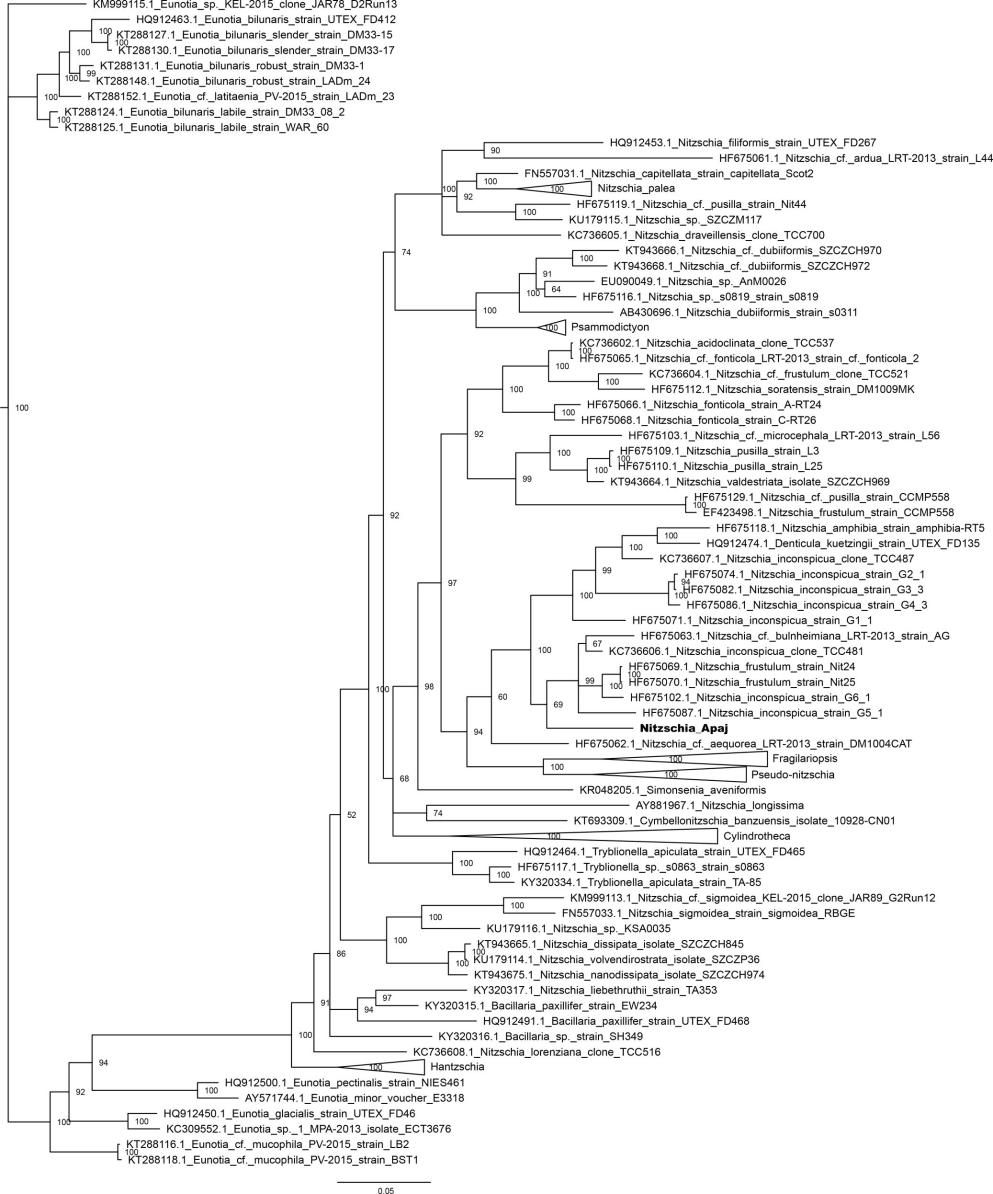
Bayesian inferred phylogenetic tree based on *rbc*L sequences. For clarity certain clades were collapsed to triangles. Posterior probability values are indicated at the nodes. Sequence acquired in this study is indicated with bold letters. Scale bar: 0.05 substitutions/site.

On the *rbc*L gene based tree (Fig 10) the new species was located in a clade formed by grade of lineages containing *N*. *amphibia*, *D*. *kuetzingii*, *N*. *inconspicua*, *N*. *supralitorea*, *N*. cf. *bulnheimiana* (Rabenhorst) H.L.Smith, *N*. *frustulum* and *N*. cf. *aequorea* Hustedt. This clade was the sister group of *Fragilariopsis* and *Pseudo-nitzschia*. *Nitzschia frustulum* and *N*. cf. *frustulum* were placed in the next lineage, while *N*. *liebetruthii* was more distantly located.

**Fig 11:**
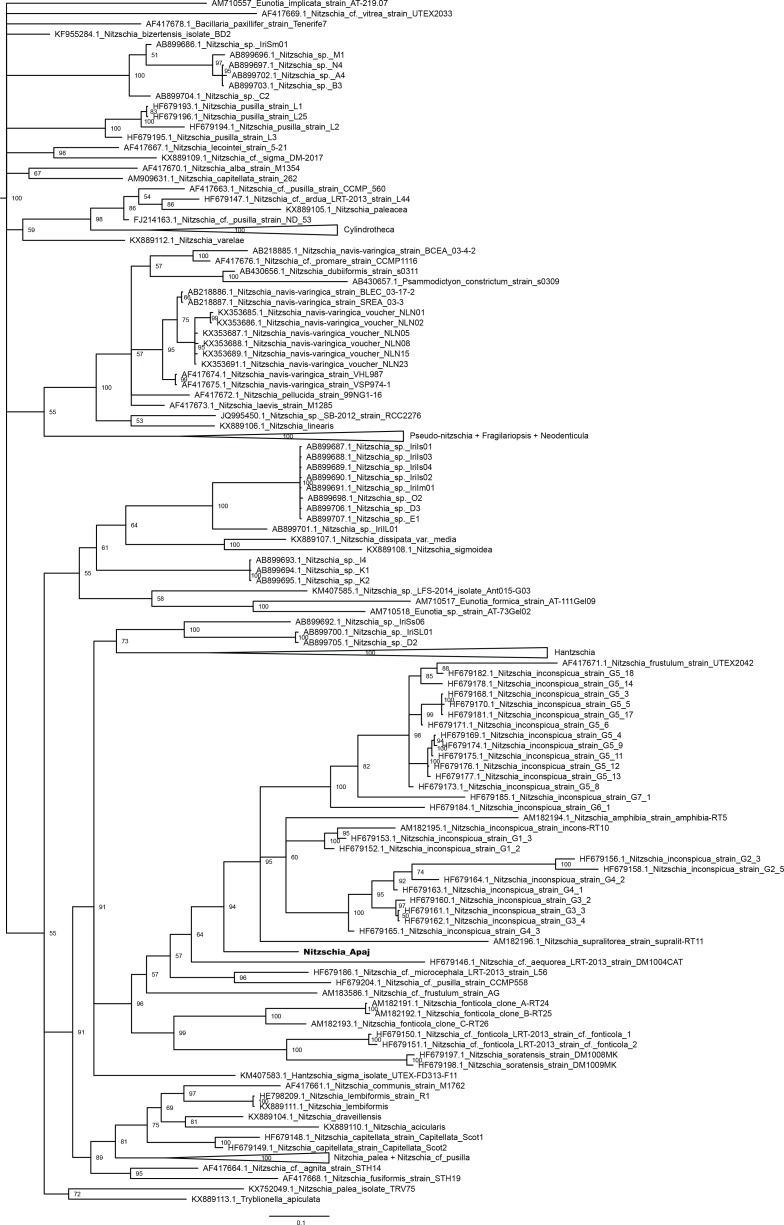
Bayesian inferred phylogenetic tree based on 28S rRNA gene sequences. For clarity certain clades were collapsed to triangles. Posterior probability values are indicated at the nodes. Sequence acquired in this study is indicated with bold letters. Scale bar: 0.1 substitutions/site.

On the 28S rRNA gene based tree (Fig 11) the clade containing the new species involved the isolates of *N*. *frustulum*, *N*. *inconspicua*, *N*. *supralitorea*, *N*. *amphibia*, *N*. cf. *aequorea*, *N*. cf. *microcephala* Grunow, *N*. cf. *pusilla*, *N*. cf. *frustulum*. This was the sister group of the clade of *N*. *fonticola* Grunow*/*cf. *fonticola* and *N*. *soratensis* E.Morales & M.L.Vis strains. The group including *Fragilariopsis* and *Pseudo-nitzschia* among others was more distantly related. The 28S rRNA gene sequence of *N*. *liebetruthii* was not available.

The uncorrected p-distance was calculated between the studied taxon and the morphologically most similar taxa. The distance was the highest in 28S rRNA and the lowest in 18S rRNA genes (Table 5). In case of 18S rRNA and *rbc*L genes, the comparison was carried out both on whole stretch and on the region proposed as barcode by Zimmermann et al. [30] and Hamsher et al. [28], respectively. The p-distance values were lower in the whole length than in the barcode region (our *rbc*L gene sequence was not fully overlapped with the *rbc*L-3P proposed by Hamsher et al. [28], the compared region was 731 nt instead of 748 nt).

**Table 5:** Uncorrected p-distance values (expressed in percentage) calculated between the new species and the taxa morphologically most similar to it.

Species	18S rRNA gene	V4 region*	28S rRNA gene	*rbc*L	*rbc*L-3P**
***Nitzschia amphibia***	2.76 (27)	5.90 (22) - 6.70 (25)	17.28 (89)	7.88 (64)	8.34 (61)
***Nitzschia bulnheimiana***	-	-	-	4.68 (38)	4.51 (33)
***Nitzschia frustulum***	7.19 (70) - 7.43 (72)	10.81 (40) -12.57 (46)	12.83 (48)– 15.64 (81)	3.94 (32) - 9.01 (73)	3.69 (27) - 9.71 (71)
***Nitzschia hantzschiana***	2.25 (22)	4.55 (17)	-	-	-
***Nitzschia inconspicua***	1.85 (18) - 2.82 (24)	4.01 (15) - 5.88 (22)	8.70 (58)– 17.59 (102)	3.94 (32) - 6.16 (50)	3.83 (28) - 6.29 (46)
***Nitzschia liebetruthii***	6.81 (66)	11.08 (41)	-	10.84 (88)	11.08 (81)
***Nitzschia supralitorea***	5.17 (44)	7.8 (29)	18.06 (95)	-	-

The numbers of different sites are in brackets.

*This comparison was limited to the region that was proposed as barcode by Zimmermann et al. [30]. The analysis also included 18S rRNA gene sequence (FR873260.1) of *Nitzschia inconspicua* from Zimmermann et al. [30].

**This comparison was limited to the region that was proposed as barcode by Hamsher et al. [28]. The analysis also included *rbc*L sequence (HQ337571.1) of *Nitzschia frustulum* strain CCMP558 from Hamsher et al. [28].

## Discussion

### Physical-chemical and benthic diatom-based biological features of the ponds

The bomb crater ponds are situated in the plain of Duna-Tisza Interfluve. This area is characterized by mosaic pattern of Solonchak (characterized by accumulation of water-soluble sodium salts mainly in the upper zones and uniform section construction without recognizable levels, [62,63]) and Solonchak-Solonetz (with accumulated water-soluble sodium salts but having definitely recognizable levels, [62,63]) soils and halophyte vegetation. In this section two groundwater flow domains were identified: a gravity-drive meteoric fresh water and an over-pressured deeper domain of saline water [64]. The groundwater level is close to the soil surface (in not more than 1 m depth) and the total dissolved salt content is high (reaches the 5000 mg L^-1^, [65]). This water causes extensive surface salinization in those discharge areas where the infiltrating freshwater does not superimpose the upwelling saline water. Where a freshwater lens is located above the ascending saline water, this fresh gravity-driven flow controls the surface distribution of salts, resulting in a mosaic pattern in the chemical variables of the surface waters beside the variation of the soil and vegetation [34].

The studied bomb craters were bordered by macrophyte belts with various widths. Based on the diatom community composition, these ponds can be rated to three types corresponding to the width of the macrophyte belt. The ponds in the “transparent” group are characterized by small water surface and broad macrophyte belt. Therefore these ponds are rarely stirred up by the wind, thus their water is transparent. The ponds in the “transitional” group have relatively large water surface and narrow marcophyte belt, hence the wind can occasionally mix them up. The ponds in the “turbid” group do not have macrophyte belts at all, their water can be stirred up by the weakest wind, so they are turbid. Regarding their physical and chemical characteristics these turbid ponds are the most similar to the soda pans of the Carpathian Basin [4].

### Ecological status of ponds based on benthic diatoms

Diatoms are widely used organisms to monitor environmental changes, because they respond to the changes of conductivity and ionic composition as well [66]. They are frequently used as ecological indicators not only in lotic but in lentic environments as well (e.g. [15,67]). Previously, the usefulness of diatoms as indicator of nutrients was investigated in many studies [68–71,16]. In case of soda ponds, the metrics indicating the changes of salt content acquire more increased role. The physical and chemical properties of the saline wetlands (e.g. pH, ionic composition, nutrient availability and light) are influenced not only by anthropogenic but also natural processes [4]. The soda pans are very important cross-continental migrating and breeding sites for aquatic birds, so they have naturally high nutrient content [61]. Therefore, trophity metric cannot be applied in case of these ponds. So, a metric which indicates the changes of the salt content had to be chosen. While increasing salt content indicates adverse processes in most water types, in soda pans the disappearance of soda character is unfavourable [22]. The diatom communities of the bomb craters showed high similarity to those natural astatic soda ponds belonging to the biological type 3 according to the Hungarian typology [33]. Hence we performed water quality assessment according to this type. In the studied area, no anthropogenic intervention has happened since the World War II bombing. So, primarily the ponds belonging to the “turbid” group can be model sites for the shallow, astatic soda lakes. The results may be useful to aid the design of soda pan restoration plans in order the soda pans to achieve the “good” ecological status corresponding to requirements of the Water Framework Directive. During their conservation, it should be managed to maintain those salinity levels that sustain their species composition, diversity and productivity. On the basis of the applied halobiont evaluation system we could distinguish the anthropogenic impact from the natural processes.

### Main drivers of the diatom composition

Altogether 80 diatom taxa, representing 33 genera, were found in our study, which is a relatively high number in such small and saline waters such as these bomb crater ponds. Bolgovics et al. [72] clearly demonstrated that the small aquatic pools can also serve favorable environments for high species richness which draws attention to the conservation biological importance of the small habitats, as well. According to Legler & Krasske [73], the diatom species composition of Van Lake was very similar to the Hungarian soda pans studied by Cholnoky [74] and the soda lakes of Burgenland studied by Legner [73].

The CCA was performed in order to assess the main environmental drivers forming the structure and the composition of diatom communities in the studied small soda ponds. The most significant variables were the salinity (and also the conductivity), pH, turbidity (and also the TSS and Secchi transparency), TP and depth. Schagerl et al. [75] found that sodium, pH and dry mass significantly contributed to the phytoplankton taxa pattern in two Kenyan soda lakes. Salinity and trophic status were found as the most important environmental variables affecting the composition of the local biota [76], while the temperature, pH, oxygen saturation, ionic composition and salinity were the main environmental variables determining the diatom composition in soda pans of the Carpathian Basin [18,20]. Our results suggest that the salinity, pH and turbidity (caused by the wind which can frequently resuspend the small particles of the soil) are the main stress factors and they are the most important environmental variables shaping the diatom communities in these bomb crater ponds.

On the basis of the diatom community composition the ponds could be divided into three distinct groups and the physical-chemical variables also markedly differed in these groups. The ponds of the “turbid” group showed the most soda features since they are characterized by high pH, conductivity, TP and TSS. The N:P ratio was very low (<1), caused by high pH, similarly to several other Hungarian soda pans [61]. The genus *Nitzschia* was represented (first of all in the “turbid” group) by the greatest number of species (16), and then the genus *Gomphonema* (dominated the “transparent” group) had the second largest number of taxa (9). *Nitzschia* species were most frequent in the saline lakes of Danube-Tisza Interfluve and Fertő-Hanság region, as well [18], and it was represented by the greatest number of species also in Mono Lake (California, USA) [77]. The dominance of the genus *Nitzschia* can be related to the high turbulence rates [78], and it was abundant in those ponds where the algae had to cope with physical stress caused by the wind-induced turbulences. Beside the *Nitzschia* taxa, the genus *Halamphora* was subdominant in “turbid” group. Probably the *Halamphora* species are shade-tolerant taxa, similarly to *Amphora* species [79]. *Nitzschia* taxa are also shade-tolerant and they are capable of feeding in heterotrophic manners [80]. In the “transparent” group the proportion of genus *Gomphonema* was the highest, followed by the genus *Achnanthidium*. They can attach in prostrate form to the substrate. In a mesocosmos experiment, the relative abundance of *Achnanthidium minutissimum* decreased with increasing of salinity [81]. Sites with low sodium ion content were characterised by high relative abundance of *Gomphonema* species in a study on epiphytic diatoms of three temporary depressional wetlands in South Africa [82].

Among the frequent species in the studied bomb crater ponds, *Anomoeoneis sphaerophora* is a worldwide distributed diatom in inland saline waters [83–88] (and it was an indicator species of Fertő-Hanság soda pans [18]). *Nitzschia vitrea* was occurred e.g. in Lake Van [68], Lake Chad area [84], Baltic Sea [89] and some soda pans of the Carpathian Basin [19].

*Gomphonema jadwigiae* Lange-Bertalot & E. Reichardt was dominant only in one pond (relative abundance 8.9%), and occurred in two other ponds but only in a few number. The species was described in 1996 as an oligotrophic indicator species [90]. We found it in three hypertrophic ponds (the total phosphorus concentrations were above 100 μg L^-1^, which is the limit of hypertrophic conditions [91], all of them were subsaline (2–2.8 g L^-1^) according to the Hammer’s [9] categorization and belonged to the “transparent” group. *Gomphonema jadwigiae* is a rare species worldwide. It was noted as an “interesting record” in the monitoring of benthic diatoms of lakes in Brandenburg [92]. It was also found in Lake Dojran and River Vardar (Republic of Macedonia), both localities are eutrophic with medium to high electrolyte content [93].

### Indication

The indicator species of the “transparent” group mostly belong to the high profile guild, first the *Gomphonema* species which prefer the low disturbance [56]. *Navicula veneta* was the most dominant diatom among the indicator species of the “transitional” group. The high percentage of the alkaliphilic *Navicula veneta* was also observed in shallow Seewinkel soda pans (Austria) by Yoshitake & Fukushima [94].

*Halamphora dominici* was one of the most dominant, frequent species and indicator of the “turbid” group in our study, which occurred in pristine, saline environments [95]. It was described from a turbid Bolivian high-mountain shallow lake with high salinity, but nowadays frequently reported from soda pans of the Carpathian Basin [19], and it is regarded as an indicator species for reference sites of soda pans of Fertő-Hanság region [20]. Among our indicator species of the “turbid” group, *Nitzschia austriaca* is a characteristic indicator species of soda waters, including the protected astatic soda pans [23]. *Nitzschia supralitorea* was found in 23 soda pans of the Carpathian Basin, generally as a dominant species [19]. *Navicula wiesneri* was found in four soda pans of the Carpathian Basin, sometimes as a dominant species [19] and we found it as indicator species of “good” ecological status in bomb crater ponds.

The mean of the relative abundance of the newly described *Nitzschia* species was the highest in the “transitional” groups, and it contributed greatly (with 11.2%) to the separation of “good” and “not-good” ecological status according to the SIMPER analysis, so we presume that the increasing abundance of this *Nitzschia* species is a signal of the degradation of the intermittent saline wetlands.

### Identification of the *Nitzschia* sp

In spite of the increasing number of publications dealing with the benthic diatoms of the Hungarian astatic soda pans [17–21], one of the most common (sometimes dominant) *Nitzschia* species has not been identified correctly yet. Therefore, we isolated it and performed sequence analysis and a detailed microscopic investigation. The new *Nitzschia* species differs from *N*. *alpina* Hustedt emend. Lange-Bertalot, *N*. *amphibia*, *N*. *annewillemsiana* Hamsher, Kopalová, Kociolek, Zidarova & Van de Vijver, *N*. *bulnheimiana*, *N*. *costei* Tudesque, Rimet & Ector, *N*. *frustulum*, *N*. *frustulum* var. *salina* Hustedt, *N*. *hantzschiana* and *N*. *inconspicua* first of all in the absence of central nodule (Table 6). *Nitzschia kahlii* Lange-Bertalot & Rumrich is smaller than the new *Nitzschia*. *Nitzschia perminuta* (Grunow) Peragallo has double-quadruple areolae at the raphe keel. One fibula is connected to one costa in *N*. *supralitorea* and *N*. *solita* Hustedt while two to three striae in the new *Nitzschia*. *Nitzschia liebetruthii* as observed from the material N°1253 (Miramar (Venezia Giulia)—Italy, "*Nitzschia liebetruthii Grun*. *et Rabh*. *Collectio Grunow 1253 'Nitzschia liebetruthii Nitzschia perpusilla'*, *in envelope Acqu*. *1901/1063*") borrowed at the Grunow Collection (David Mann, personal communication) contain cells that are typically lanceolate with the raphe interrupted at the central nodule. *Nitzschia liebetruthii* cells are linear-lanceolate to lanceolate and larger cells are slightly flexed (as might be expected in post-initial cells) thus slightly constricted in larger frustules. The newly described *Nitzschia* species closely resembles *Nitzschia frustulum*, which is regarded as an extremely salt tolerant species but it could become abundant in brackish waters because it has a competition advantage over other species [85], and it is tolerant to fluctuations of osmotic pressure [96]. *Nitzschia frustulum* was found in permanent salt evaporation ponds in Spain [97] and in East African saline lakes [85]. It is characteristic to all of Hungarian perennial soda lakes Fertő, Velence and Szelidi [78,87]. It is the most frequent species in Central European astatic soda pans [19] and the second most frequent in North American saline lakes [86].

**Table 6:** Morphological features of new species and some similar *Nitzschia* taxa.

Species	Length (μm)	Width (μm)	Stria / 10 μm	Fibulae / 10 μm	Areolae	Pattern of areolae at the keel	Raphe	Central nodule	Transapical costae merging with a fibula	References
***Nitzschia* nov. sp.**	17–27	2.4–3.7	19–30	9–12	visible in LM, the last one is elliptical, uniseriate striae	single, thickened ring around them	continuous	no	two-three	this study
***N*. *alpina* Hustedt emend. Lange-Bertalot**	8–35	3–4	23–27	10–14	relatively coarse, appear distinctly in LM by [98] but indistinctly by [99]	double	interrupted	yes	one	[98–100]
***N*. *amphibia* Grunow**	6–50	4–6	13–18,	7–9	visible in LM, uniseriate striae	double	interrupted	yes	one-two	[98]
***N*. *annewillemsiana* Hamsher, Kopalová, Kociolek, Zidarova & Van de Vijver**	10–20	3–4	24–26	11–12	visible in LM, uniseriate striae	double, occasionally quadruple	interrupted	distinct central nodule present on some specimens	two-three	[101]
***N*. *bulnheimiana* (Rabenhorst) H.L.Smith**	12–60	4–4.7	19–22	8–13	easy resolvable	regularly double	interrupted	yes		[98,102]
***N*. *costei* Tudesque, Rimet & Ector**	8–45	2.5–4.5	23–27	(7)9-12(13)	visible in LM, uniseriate striae	always double	interrupted	yes	two-three	[102]
***N*. *frustulum* (Kützing) Grunow in Cleve & Grunow**	10.8–34	3–3.9	27–30	13–15	uniseriate striae	single	interrupted	yes	rarely one- more frequent two	[103]
***N*. *frustulum* var. *subsalina* Hustedt**	8–16.8	2.4–2.9	25.1–27.8	10.1–15	uniseriate striae	single	interrupted	yes	rarely one- more frequent two	[103]
***N*. *hantzschiana* Rabenhorst**	8–50	3–5	20–26	7–13	relatively coarse and appear distinctly	double- quadruple	interrupted	yes	two-three	[25,98]
***N*. *inconspicua* Grunow**	6–11.5	2.6–3.1	23.7–28.7	10.6–17	visible, uniseriate striae	single	interrupted	yes	two-three	[103,104]
***N*. *kahlii* Lange-Bertalot & Rumrich**	8–12	2.1–2.8	24–26	11–13	not easy to resolve	single	continuous	no	two	[105,106]
***N*. *liebetruthii* Rabenhorst**	14–32	2.8–3.2	23–25	12–14	rather easily resolvable	single	interrupted	yes	no data	[98,106,107]
***N*. *perminuta* (Grunow) Peragallo**	8–45	2.5–3	26–36	10–16	usually detectable with LM	double-quadruple	continuous	no	two	[25,98,100,108]
***N*. *solita* Hustedt**	18–50	4–6	24–28	11–16	not easy to resolve in LM, uniseriate striae	single	continuous	no	one-two	[25,98]
***N*. *supralitorea* Lange-Bertalot**	10–25	2.5–4	25–34	14–20	not clearly visible in LM, uniseriate striae	single	continuous	no	(one-)two	[19,25,98]
***N*. *dentatum* Suriyanti & Usup**	17.0–18.0	2.5–4.5	70–78	11–13	uniseriate striae	single	continuous	no	undefinable	[109]
***N*. *johorensis* Suriyanti & Usup**	7.1–11.8	1.8–3.5	30–33	11–12	uniseriate striae	single	continuous	no	one-two	[109]
***N*. *kociolekii* Alakananda, B.Karthick, J.C.Taylor & P.B.Hamilton**	14.0–32.5	4–5	16–19	6–8	unequally spaced, uniseriate striae	double	discontinuous	gap at the central area	(one-)two	[110]
***N*. *tripudio* Alakananda, B.Karthick, J.C.Taylor & P.B.Hamilton**	12.5–19.0	2.5–3	24–26	6–10	lip-shaped areolae, uniseriate striae	areolae at keel are composed of three linear slits that intersect	continuous	no	two-three	[110]

Since the newly described *Nitzschia* is very similar to *Nitzschia frustulum*, it is possible that some of the previous *N*. *frustulum* occurrences were actually this new species. The main difference is that the new species does not have a central nodule while *N*. *frustulum* does. The other remarkable difference is in the number of fibulae (*N*. *frustulum* has 13–15 fibulae in 10 μm while the new *Nitzschia* species has a maximum of 12).

Based on available DNA sequence information of 18S and 28S rRNA as well as *rbc*L genes, the following species appeared to be close relatives of the studied taxon: *Nitzschia amphibia*, *N*. *inconspicua*, *N*. *frustulum*, *N*. *supralitorea* and *Denticula kuetzingii*. *Nitzschia hantzschiana* was also closely related, however, only 18S rRNA gene sequence was available from this species. These *Nitzschia* species that belong to the section Lanceolatae were also similar to our species according to light and electron microscopy features of the frustule. Close relatedness of *D*. *kuetzingii* to *N*. *amphibia* was also shown by other authors (e.g. [104,111]).

The sequences of the three genes were rarely obtained from the same strains of a species, making multi-gene analyses difficult. Moreover, some isolates could not be exactly identified (e.g. *Nitzschia* cf. *frustulum*) or even may be misidentified like in the case of *N*. *frustulum* UTEX 2042 strain that was referred as *N*. *inconspicua* by Rovira et al. [104] because of morphometric measurements and valve morphology. This contributes to the non-monophyly of some species.

Our phylogenetic analyses proved that *Nitzschia* genus is not monophyletic as was shown previously based on all of the three studied genes [104,111,112]. The mentioned authors could define major clades of *Nitzschia* based on 18S rRNA [112] as well as 28S rRNA and *rbc*L [104,111] genes. According to them the *Nitzschia* section Lanceolatae formed two groups: *N*. *inconspicua*, *N*. *frustulum*, *N*. *supralitorea*, *N*. *amphibia*, *N*. *fonticola* were closely related to *Fragilariopsis* and *Pseudo-nitzschia* (subclade 2b within the clade 2 in the maximum likelihood tree prepared using secondary structure alignment in Rimet et al. [112], group/clade I. in Rovira et al. [104] and Carballeira et al. [111]) and separated from *N*. *palea* and related species (subclade 1c within the clade 1 in Rimet et al. [112], group/clade III in Rovira et al. [104] and Carballeira et al. [111]). Our phylogenetic trees also showed similar groupings of the *Nitzschia* section Lanceolatae species, and the new species from Apaj belonged to the first group.

On all phylogenetic trees, our species showed clear separation from other taxa. This was supported by relatively high p-distance values. Strains of *Nitzschia inconspicua* differed least from it.

The divergence between species varied according to the genes. The 18S rRNA gene showed the lowest differences corresponding to the previous findings by several authors (e.g. [113,114]). Because of the low divergence of the whole stretch, Zimmermann et al. [30] proposed a shorter region for barcode marker containing V4 subregion, the largest and the most complex of the highly variable regions within the 18S locus. The authors found 1.3–21.2% (average: 9.3%) interspecific distance in this barcode region of the studied *Nitzschia* species, however, they involved taxa from different lineages (e.g. *N*. *inconspicua* and *N*. *palea*). Luddington et al. [115] successfully applied a 2% threshold for this marker to the separate species; however, they used centric taxa. Nevertheless, our species showed more than 2% divergence in this region from the similar taxa.

Hamsher et al. [28] studied *rbc*L and 28S rRNA genes in comparison with other markers (*cox*1 and universal plastid amplicon). They proposed a 748 bp long part at the 3’ end of *rbc*L (*rbc*L-3P) as a primary and D2-D3 region of the 28S rRNA gene as a secondary barcoding marker. The former marker could distinguish the most closely related ones of the studied taxa (these were *Sellaphora* isolates): the distance was 0.14% (2 bp) in the whole *rbc*L, 0.1–0.3% (1–2 bp) in *rbc*L-3P. Although rRNA gene D2/D3 region of the 28S rRNA gene could not resolve the species pair that *rbc*L-3P could, it could separate 96% of the studied species. The lowest distance was 0.3% (2 bp) in it. Based on the investigation of CCMP isolates the authors concluded that 1 bp difference in *rbc*L-3P and D2/D3 region of the 28S rRNA gene could indicate intraspecific variation. Our species showed much higher difference from the closely related species both in *rbc*L, *rbc*L-3P and 28S rRNA genes.

The sequence analysis results of the three markers suggest that the studied taxon from Apaj had differences from the published sequences of species of Bacillariaceae were high enough; therefore, it could be distinguished from them as a separate species.

#### New *Nitzschia* species description

*Nitzschia reskoi* Ács, Duleba, C.E.Wetzel & Ector

Valve length 17–27 μm, width 2.4–3.7 μm, 19–30 striae in 10 μm, 9–12 fibulae in 10 μm. In SEM, externally the striae are uniseriate and are never doubled at the raphe keel. Areolae are irregular, circular, elliptical or sometimes missing within a stria. The first areola in the stria (near the raphe) has a thickened ring around them and the last one is elliptical. On the mantle, the striae are very short, comprising two areola, one of them is covered by hymen internally. Externally the distal raphe fissures hooked, internally terminating in small helictoglossae. The central nodule absent.

The species is frequent in Hungarian saline lakes (both in astatic and perennial ones) and soda pans and presumably widespread in other inland saline lakes, but due to the uncertainity of its taxonomical position it has probably been identified occasionally as *Nitzschia frustulum*, despite the lack of central nodule. *Nitzschia reskoi* prefers turbid, hypertrophic soda waters.

Holotype: MTA-ÖK-14/12, deposited at the Centre for Ecological Research, Danube Research Institute, Budapest, Hungary Fig 8C, R. Epiphyton collected from pond N^o^13 on 8/05/2014.

Type locality: Apaj, Hungary (47°7.403'N 19°8.187'E).

Isotypes: HNHM-ALG-2261 deposited at the Hungarian Natural History Museum, Budapest, Hungary.

Etymology: The name is in honor of Dr. Mária Reskóné Nagy, an Hungarian hydrobiologist who worked for many years on Lake Velence, an Hungarian soda lake.

#### Distribution of *Nitzschia reskoi*

**Fig 12:**
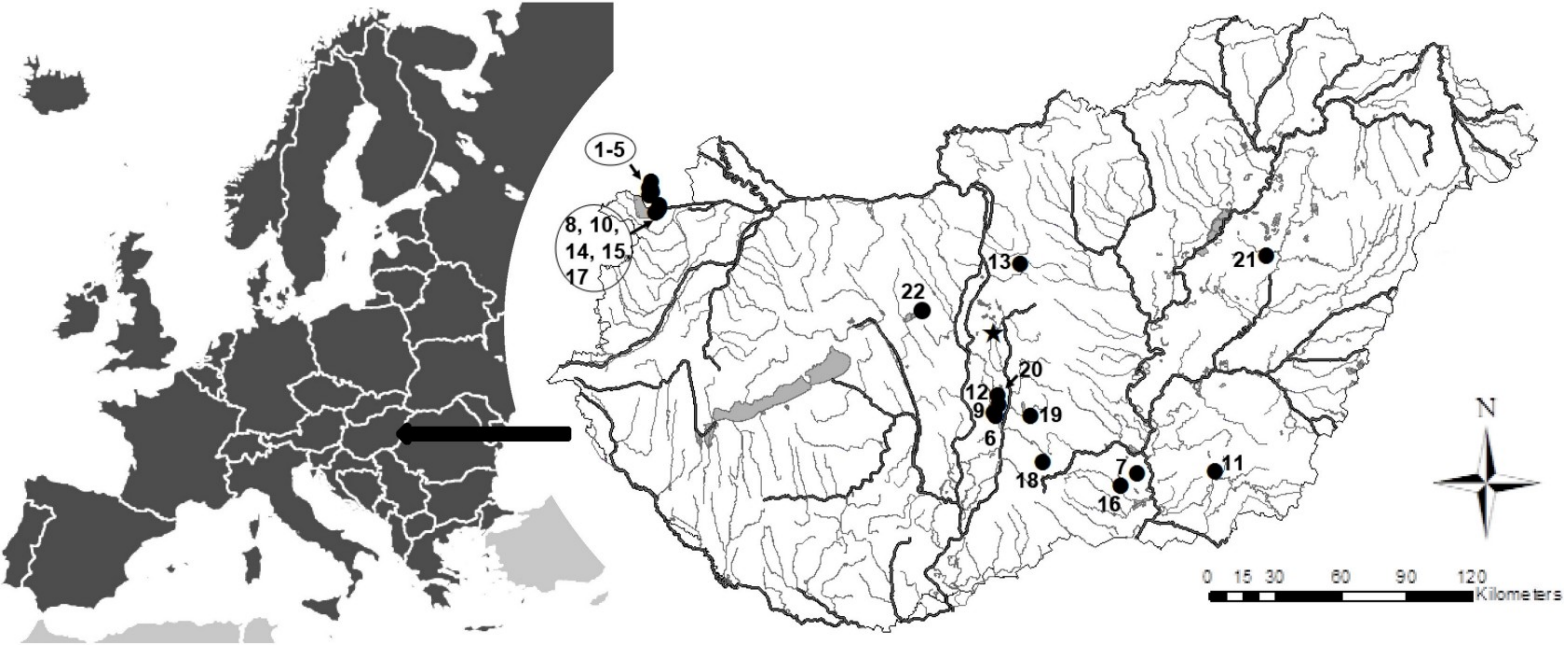
Distribution of *Nitzschia reskoi*. Star = type locality. See numbers in the text.

Occurrences of *N*. *reskoi* in Carpathian Basin are illustrated in Fig 12. In Hungary we have found *N*. *reskoi* in bomb crater ponds of Kiskunság National Park near Apaj (Fig 12), bomb crater ponds of Hortobágy National Park near Nagyiván (indicated with 21 in Fig 12) and Lake Velence (22). Stenger-Kovács & Lengyel [19] published its occurrence (identified as *Nitzschia frustulum* according to their micrographs) in the following aquatic systems: Bába-szék (indicated with 6 in Fig 12), Bíbic-tó (7), Borsodi-dűlő (8), Böddi-szék (9), Cikes (10), Kardoskúti Fehér-tó (11), Kelemen-szék (12), Kisréti-tó (13), Legény-tó (14), Nyéki-szállás (15), Ősze-szék (16), Pap-rét (17), Pirtói Nagy-tó (18), Sárkány-tó (19), Zab-szék (20). Moreover it has occurred in other soda pans of Carpathian Basin published in Stenger-Kovács & Lengyel [19] as *Nitzschia frustulum*: Albersee (1), Herrnsee (2), Kirchsee (3), Untersee (4), Zicklacke (5).

## Conclusions

1. The community-level response of epiphytic diatoms to natural environmental stress could be detected with non-parametric multidimensional scaling. The macrophyte belt determined the turbidity and transparency, therefore, we distinguish three different types: “turbid”, “transparent” and “transitional” ponds, which had different salinity, pH and TSS content that effected the composition of the epiphytic diatom communities. The “transparent” group was dominated by typical freshwater species mainly attaching with stalk, while in the “turbid” group adnate and motile halophylic diatoms prevailed. The “transitional” group was dominated by erected, adnate and motile forms as well.
2. Epiphytic diatoms proved to be applicable in assessment of the ecological status of astatic soda ponds. Ponds in “good” and “not-good” status differing significantly in their environmental variables showed clear separation according to their epiphytic diatom community composition. This difference could be revealed in traits of diatoms: the “good” status could be characterised with higher proportion of motile or adnate, micro cell-sized diatoms with medium oxygen requirements and heterotrophic N-uptake strategy.
3. We identified indicator species of the three types of ponds according to the width of macrophyte belt. The “transparent” type was indicated by *Gomphonema* species (*G*. *angustatum*, *G*. *affine* Kützing, *G*. *clavatum*, *G*. *micropus*, *G*. *paludosum* E. Reichardt) and *Achnanthidium minutissimum*. Two *Nitzschia* species, *N*. *liebetruthii* and the newly described species as well as *Navicula veneta* and *Psammodictyon constrictum* were related to the “transitional” group. Dominance of *Halamphora dominici*, *Nitzschia austriaca*, *N*. *supralitorea*, *Navicula wiesneri* and *N*. *radiosa* Kützing characterized the “turbid” group.
  Indicator species of “good” and “not-good” ecological status could be also determined. *Halamphora dominici*, *Navicula wiesneri*, *Nitzschia austriaca*, *N*. *supralitorea* indicated the “good” ecological status while *A*. *minutissimum*, *Craticula buderi*, *G*. *angustatum*, *G*. cf. *micropus*, *G*. *clavatum*, *Nitzschia acidoclinata*, *N*. *commutata*, *N*. *palea* and *P*. *constrictum* were related to the “not-good” status. The diatoms indicating the “not-good” ecological status are typical freshwater species. In most water types the increasing salt content indicates deterioration processes, in soda pans the disappearance of soda character is disadvantageous, so here we should choose a metric that indicates the changes of the salt content.
4. We described a species new to science. This *Nitzschia* species was the most abundant in the ‘transitional’ group. Our results suggest that the increasing dominance of this species indicates deterioration of the soda character.

According to Reese et al. [116], a true “extreme” environment on Earth would be hot brine having high pH. Some of the studied bomb crater ponds (first of all the members of “turbid” group) had high salinity, pH and turbidity, their waterbody could warm up and the suspended matter content was extremely high, so the light availability for the algae was very low. Following the proposed terminology of Reese et al. [116], we can say that these ponds are really extreme habitats and the indicator diatoms of these ponds are true “boundary organisms”. These results highlight the importance of studies finding rules that explain the composition and abundance of coexisting species, which is a central issue of community ecology.

These saline bomb crater ponds are good inference models for intermittent soda pans of the Carpathian Basin because they share many common features (physical-chemical variables and diatom composition as well) with the Central European astatic soda pans. To define reference conditions and select their variations due to natural stress is a great challenge of ecological status assessment. On the studied area, no human intervention has occurred since the World War II bombing, because it belongs to a national park. Therefore, primarily the ponds belonging to the "turbid" group can be model sites for the shallow, astatic soda lakes. Their indicators are mainly the species *Nitzschia austriaca* and *Halamphora dominici*, which can reach strong dominance in the phytobenthos. The increasing abundance of *Nitzschia reskoi* seems to be a signal of losing sodic character of the intermittent saline wetlands. Our results may be used to design soda pan restoration plans in order for the soda pans to achieve the “good” ecological status corresponding to the requirements of the Water Framework Directive.

## Supporting information

S1 Table Construction of the combined traits. (DOCX)

S2 Table The results of the ecological status assessment of bomb crater ponds. H = high, G = good, M = moderate, P = poor, B = bad. The ponds were grouped according to the macrophyte belt: 1 = “transparent”, 2 = “transitional”, 3 = “turbid”. EQR: ecological quality ratio.
(DOCX)

S3 Table The average relative abundance of the dominant taxa in the three groups and their frequency. (DOCX)

S1 Fig Typical pictures of bomb crater ponds of “transparent” (A), “transitional” (B) and “turbid” (C) groups.
(TIF)
