# Supplementary material for: Unexpected consequences of bombing. Community level response of epiphytic diatoms to environmental stress in a saline bomb crater pond area
Source: PLoS One. 2018 Oct 25;13(10):e0205343. doi: 10.1371/journal.pone.0205343 (PMC6201898; doi:10.1371/journal.pone.0205343)
Supplement: S1 Table — (DOCX) [file pone.0205343.s001.docx]

**S1 Table. Construction of the combined traits.**

| **Code of combined traits** | **Attachment to the surface** | **Nutrient demand** | **Resistance to physical disturbances** | **~ guild (according to Rimet & Bouchez 2012)** |
| --- | --- | --- | --- | --- |
| CT1 | strong (adnate) | low | high | low profile |
| CT 2 | weak (erected) | high | low | high profile |
| CT 3 | weak (motile) | high | low | motile |
| CT 4 | no (planktic) | - | low | planktic |
