# Supplementary material for: Unexpected consequences of bombing. Community level response of epiphytic diatoms to environmental stress in a saline bomb crater pond area
Source: PLoS One. 2018 Oct 25;13(10):e0205343. doi: 10.1371/journal.pone.0205343 (PMC6201898; doi:10.1371/journal.pone.0205343)
Supplement: S2 Table — H = high, G = good, M = moderate, P = poor, B = bad. The ponds were grouped according to the macrophyte belt: 1 = “transparent”, 2 = “transitional”, 3 = “turbid”. EQR: ecological quality ratio. (DOCX) [file pone.0205343.s002.docx]

**S2 Table. The results of the ecological status assessment of bomb crater ponds.**

| **No. of ponds** | **Group** | **Quality** | **EQR** |
| --- | --- | --- | --- |
| 4 | 2 | G | 0,72 |
| 6 | 1 | B | 0,08 |
| 7 | 1 | B | 0,05 |
| 8 | 1 | B | 0,17 |
| 9 | 1 | B | 0,09 |
| 10 | 2 | M | 0,59 |
| 11 | 2 | P | 0,36 |
| 12 | 2 | G | 0,66 |
| 13 | 2 | G | 0,71 |
| 14 | 2 | G | 0,77 |
| 15 | 1 | B | 0,01 |
| 17 | 2 | M | 0,52 |
| 19 | 1 | B | 0,12 |
| 21 | 2 | H | 0,80 |
| 22 | 1 | B | 0,18 |
| 24 | 2 | G | 0,73 |
| 29 | 2 | P | 0,39 |
| 34 | 2 | B | 0,17 |
| 39 | 1 | B | 0,07 |
| 42 | 2 | P | 0,22 |
| 45 | 2 | M | 0,56 |
| 46 | 2 | P | 0,36 |
| 47 | 1 | B | 0,16 |
| 48 | 1 | P | 0,36 |
| 51 | 1 | P | 0,25 |
| 52 | 2 | G | 0,71 |
| 54 | 1 | P | 0,30 |
| 56 | 3 | M | 0,56 |
| 57 | 2 | G | 0,70 |
| 58 | 3 | G | 0,67 |
| 65 | 3 | G | 0,67 |
| 66 | 3 | G | 0,62 |
| 69 | 2 | B | 0,18 |
| 74 | 2 | G | 0,61 |
| 77 | 2 | M | 0,59 |
| 78 | 2 | G | 0,66 |
| 80 | 3 | G | 0,60 |
| 83 | 2 | P | 0,34 |
| 84 | 3 | G | 0,78 |
| 91 | 2 | M | 0,56 |
| 93 | 2 | P | 0,36 |
| 94 | 2 | B | 0,07 |
| 96 | 2 | P | 0,30 |
| 97 | 1 | B | 0,16 |
| 99 | 2 | P | 0,22 |
| 102 | 2 | G | 0,69 |
| 104 | 2 | G | 0,79 |
| 111 | 3 | G | 0,77 |

H = high, G = good, M = moderate, P = poor, B = bad. The ponds were grouped according to the macrophyte belt: 1 = “transparent”, 2 = “transitional”, 3 = “turbid”. EQR: ecological quality ratio.
