# Supplementary material for: Unexpected consequences of bombing. Community level response of epiphytic diatoms to environmental stress in a saline bomb crater pond area
Source: PLoS One. 2018 Oct 25;13(10):e0205343. doi: 10.1371/journal.pone.0205343 (PMC6201898; doi:10.1371/journal.pone.0205343)
Supplement: S3 Table — (DOCX) [file pone.0205343.s003.docx]

**S3 Table. The average relative abundance of the dominant taxa in the three groups and their frequency.**

| **Taxa** | **Abbreviation** | **Transparent (1)** | **Transitional (2)** | **Turbid (3)** | **Frequency (%)** |
| --- | --- | --- | --- | --- | --- |
| *Achnanthidium minutissimum* (Kützing) Czarnecki | ADMI | 17,5 | 1,0 | 0,0 | 33,3 |
| *Craticula buderi* (Hustedt) Lange-Bertalot | CRBU | 1,5 | 0,0 | 0,0 | 37,5 |
| *Craticula halophila* (Grunow ex Van Heurck) D.G.Mann | CHAL | 0,0 | 0,6 | 0,0 | 4,2 |
| *Eunotia bilunaris* (Ehrenberg) Mills var. *bilunaris* | EBIL | 0,0 | 0,2 | 0,0 | 6,3 |
| *Gomphonema angustatum* (Kützing) Rabenhorst | GANG | 40,2 | 6,1 | 0,0 | 72,9 |
| *Gomphonema* cf. *micropus* Kützing | GMIC | 3,7 | 2,4 | 0,0 | 56,3 |
| *Gomphonema clavatum* Ehrenberg | GCLA | 3,4 | 0,0 | 0,0 | 29,2 |
| *Gomphonema jadwigiae* Lange-Bertalot & E.Reichardt | GJAD | 0,0 | 0,4 | 0,0 | 6,3 |
| *Gomphonema paludosum* E.Reichardt | GPLD | 2,7 | 0,0 | 0,0 | 8,3 |
| *Gomphonema parvulum* Kützing | GPAR | 0,0 | 0,2 | 0,0 | 2,1 |
| *Halamphora dominici* Ács & Levkov | HDOM | 0,0 | 11,9 | 35,2 | 87,5 |
| *Halamphora paraveneta* (Lange-Bertalot, Cavacini, Tagliaventi & Alfinito) Levkov | HPVE | 0,0 | 0,0 | 2,1 | 33,3 |
| *Navicula recens* (Lange-Bertalot) Lange-Bertalot | NRCS | 0,0 | 0,7 | 0,0 | 41,7 |
| *Navicula veneta* Kützing | NVEN | 15,4 | 34,9 | 10,3 | 100,0 |
| *Navicula wiesneri* Lange-Bertalot | NWIE | 0,0 | 3,2 | 4,9 | 72,9 |
| *Nitzschia acidoclinata* Lange-Bertalot | NACD | 0,0 | 1,0 | 0,0 | 35,4 |
| *Nitzschia austriaca* Hustedt | NAUS | 0,0 | 3,2 | 32,1 | 64,6 |
| *Nitzschia reskoi* sp. nov. | NIRE | 4,8 | 13,8 | 3,5 | 100,0 |
| *Nitzschia* cf. *liebetruthii* Rabenhorst | NLBT | 2,6 | 9,4 | 0,0 | 81,3 |
| *Nitzschia pusilla* (Kützing) Grunow emend. Lange-Bertalot | NIPU | 0,0 | 0,0 | 1,9 | 10,4 |
| *Nitzschia supralitorea* Lange-Bertalot | NZSU | 0,0 | 2,1 | 8,1 | 47,9 |
| *Psammodictyon constrictum* (W.Gregory) D.G.Mann in Round et al. | PCON | 1,9 | 4,7 | 0,0 | 68,8 |
| *Tabularia fasciculata* (C.Agardh) D.M.Williams & Round | TFAS | 1,0 | 0,0 | 0,0 | 10,4 |
| *Tryblionella hungarica* (Grunow) D.G.Mann | THUN | 0,0 | 0,8 | 0,0 | 33,3 |
| other | other | 5,3 | 3,5 | 1,8 |  |
