# Supplementary figures and images for: Unexpected consequences of bombing. Community level response of epiphytic diatoms to environmental stress in a saline bomb crater pond area
Source: PLoS One. 2018 Oct 25;13(10):e0205343. doi: 10.1371/journal.pone.0205343 (PMC6201898; doi:10.1371/journal.pone.0205343)

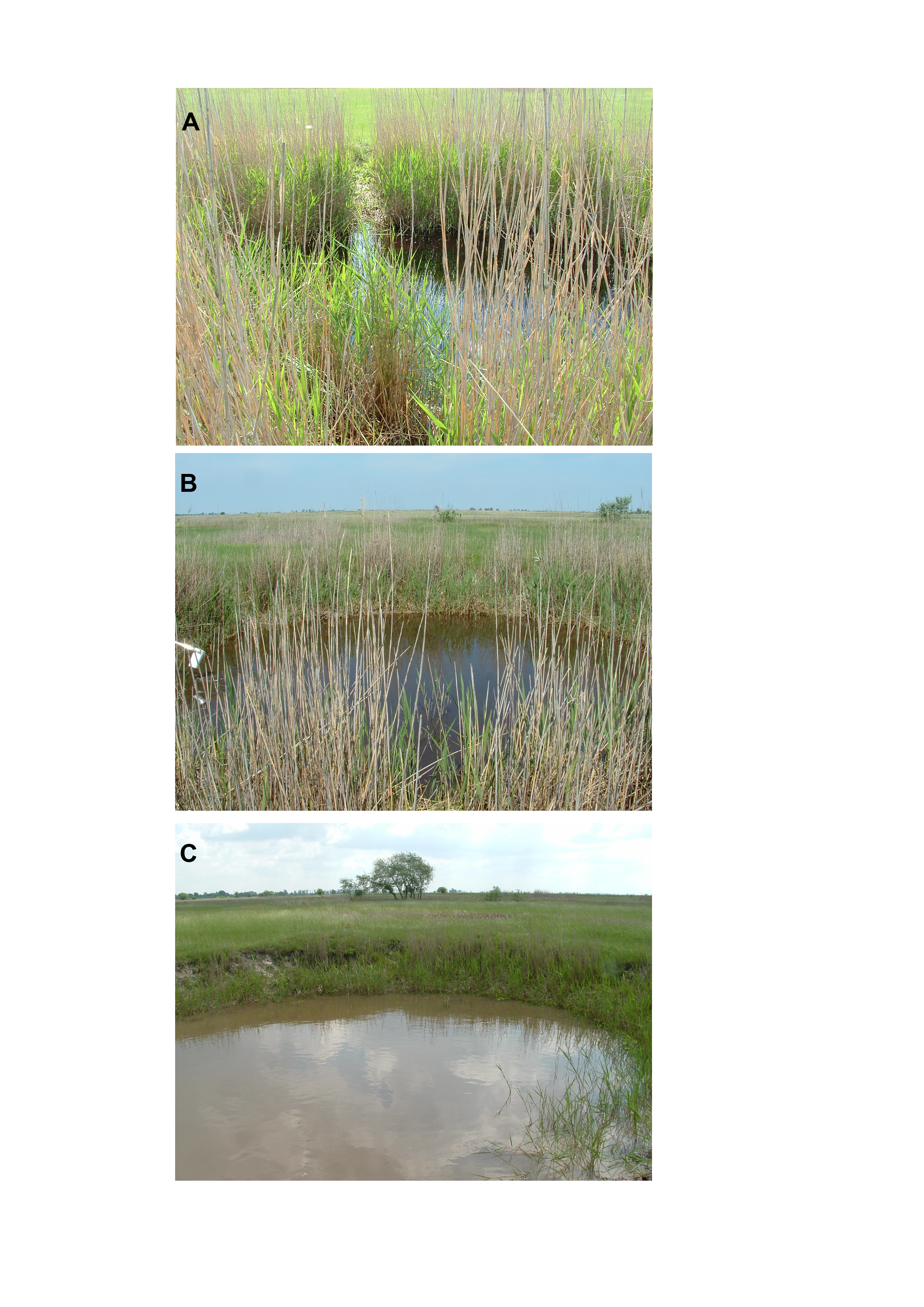

Supplement: S1 Fig — Typical pictures of bomb crater ponds of “transparent” (A), “transitional” (B) and “turbid” (C) groups. (TIF) [file pone.0205343.s004.tif]
